# Feasibility of Progressive Strength Training Implemented in the Acute Ward after Hip Fracture Surgery

**DOI:** 10.1371/journal.pone.0093332

**Published:** 2014-04-03

**Authors:** Lise Kronborg, Thomas Bandholm, Henrik Palm, Henrik Kehlet, Morten Tange Kristensen

**Affiliations:** 1 Physical Medicine and Rehabilitation Research – Copenhagen (PMR-C), Copenhagen University Hospital, Hvidovre, Denmark; 2 Department of Physical Therapy, Copenhagen University Hospital, Hvidovre, Denmark; 3 Clinical Research Centre, Copenhagen University Hospital, Hvidovre, Denmark; 4 Department of Orthopedic Surgery, Copenhagen University Hospital, Hvidovre, Denmark; 5 Section for Surgical Pathophysiology, Rigshospitalet, Copenhagen University, Copenhagen, Denmark; Delft University of Technology (TUDelft), Netherlands

## Abstract

**Importance:**

Patients with a hip fracture lose more than 50% knee-extension strength in the fractured limb within one week of surgery. Hence, immediate progressive strength training following hip fracture surgery may be rational, but the feasibility unknown.

**Objective:**

To examine the feasibility of in-hospital progressive strength training implemented in the acute ward following hip fracture surgery, based on pre-specified criteria for feasibility.

**Design, Setting and Patients:**

A prospective cohort study conducted in an acute orthopedic hip fracture unit at a university hospital. A consecutive sample of 36 patients, 18 with a cervical and 18 with a trochanteric hip fracture (27 women and 9 men, mean (SD) age of 79.4 (8.3) years) were included between June and December 2012.

**Intervention:**

A daily (on weekdays) program of progressive knee-extension strength training for the fractured limb, using ankle weight cuffs in 3 sets of 10 repetition maximum loadings.

**Main outcomes and Measures:**

The primary outcome was the change in training load (kg) during the knee-extension strength training. The secondary outcomes were changes in hip fracture-related pain and maximal isometric knee-extension strength.

**Results:**

The strength training was commenced at a mean of 2.4 (0.7) days after surgery. The training loads (kilograms lifted) increased from 1.6 (0.8) to 4.3 (1.7) kg over 4.3 (2.2) training sessions (*P*<.001). The maximal isometric knee-extension strength of the fractured limb increased from 0.37 (0.2) to 0.61 (0.3) Nm/kg (*P*<.001), while the average strength deficit in the fractured limb decreased from 50% to 32% (% non-fractured, *P*<.001). Only 3 of 212 sessions were not performed because of severe hip fracture-related pain.

**Conclusion and Relevance:**

Progressive knee-extension strength training of the fractured limb commenced in the acute ward seems feasible, and may reduce strength asymmetry between limbs without hip pain interfering. The clinical efficacy needs confirmation in a randomized controlled design.

**Trial Registration:**

ClinicalTrials.gov ID: NCT01616030

## Introduction

Factors affecting the functional prognosis after hip fracture surgery are multiple [1], and patients are at risk of decreased physical function [2]–[4], new injurious falls and fractures [5], [6], and increased need of supportive care [7]. Despite the fact that patients follow a multimodal fast-track program [8], a loss of knee-extension strength in the fractured limb of more than 50% (% of non-fractured) occurs during the first week after hip fracture surgery and is associated with impaired physical function [9]. Corresponding deficits are also reported within 2–3 weeks following hip fracture surgery [10], [11]. Patients with trochanteric fractures seem to experience greater knee-extension strength deficits, perform worse in functional parameters, and experience more fracture-related pain compared to patients with cervical fractures [9], [12]–[15], which indicates fracture type-specific pathophysiology.

With respect to recovery after a hip fracture, early mobilization [16]–[18] and extended physical therapy including strength training implemented 6 weeks after fracture seem to promote recovery of physical function [19]. Still, “current guidelines do not include detailed recommendations about exercise after hip fracture” [20] and no study has yet succeeded in a full recovery of the early loss of knee-extension strength [10], [11], [21], [22] or function [22], [23]. In theory, physical therapy including strength training should optimally be implemented in the acute ward immediately following surgery, where the deficits are greatest, to avoid further functional decline and to substantially enhance recovery after a hip fracture. However, only two studies [10], [22] have investigated strength training shortly (2 weeks) following the fracture. Although elective surgery and hip fractures are not the same, preliminary evidence suggests that strength training implemented immediately following total hip [24] and knee [25] arthroplasty is feasible and does not appear to exacerbate postoperative symptoms. If this is also true for patients following a hip fracture is currently unknown.

The primary aim of this study was to examine the feasibility of in-hospital progressive strength training implemented immediately following hip fracture surgery, based on pre-specified criteria for feasibility. We decided that feasibility was indicated if: 1) the absolute training loads increased progressively, 2) fracture-related pain during strength training did not increase during the program, and 3) more than 80% of the planned training sessions were completed.

## Methods

### Design and Setting

The study was a prospective cohort study performed as a feasibility study [26] in an acute orthopedic hip fracture unit at a university hospital. The main purpose was to indicate the feasibility of the strength training intervention prior to the confirmatory study registered at clinicaltrials.gov (NCT00848913). The protocol for this trial and supporting CONSORT checklist are available as supporting information; see Checklist S1 and Protocol S1.

After written informed consent, patients were to strength train their knee extensors in the fractured limb progressively on all weekdays throughout their hospitalization. The same skilled physical therapist, who was blinded to all baseline data until end of study, allocated all eligible patients for the present study and supervised all test and training sessions, except for one test (1% of tests) and four (2%) training sessions. The study was approved by the Capital Region’s Research Ethics Committee (H-A-2007-0127), registered at clinicaltrials.gov (NCT01616030), and conducted according to the principles expressed in the Declaration of Helsinki. The reporting of the study adheres to the STROBE guidelines for cohort studies [27], (Please see Checklist S1).

### Patients

**Figure 1:**
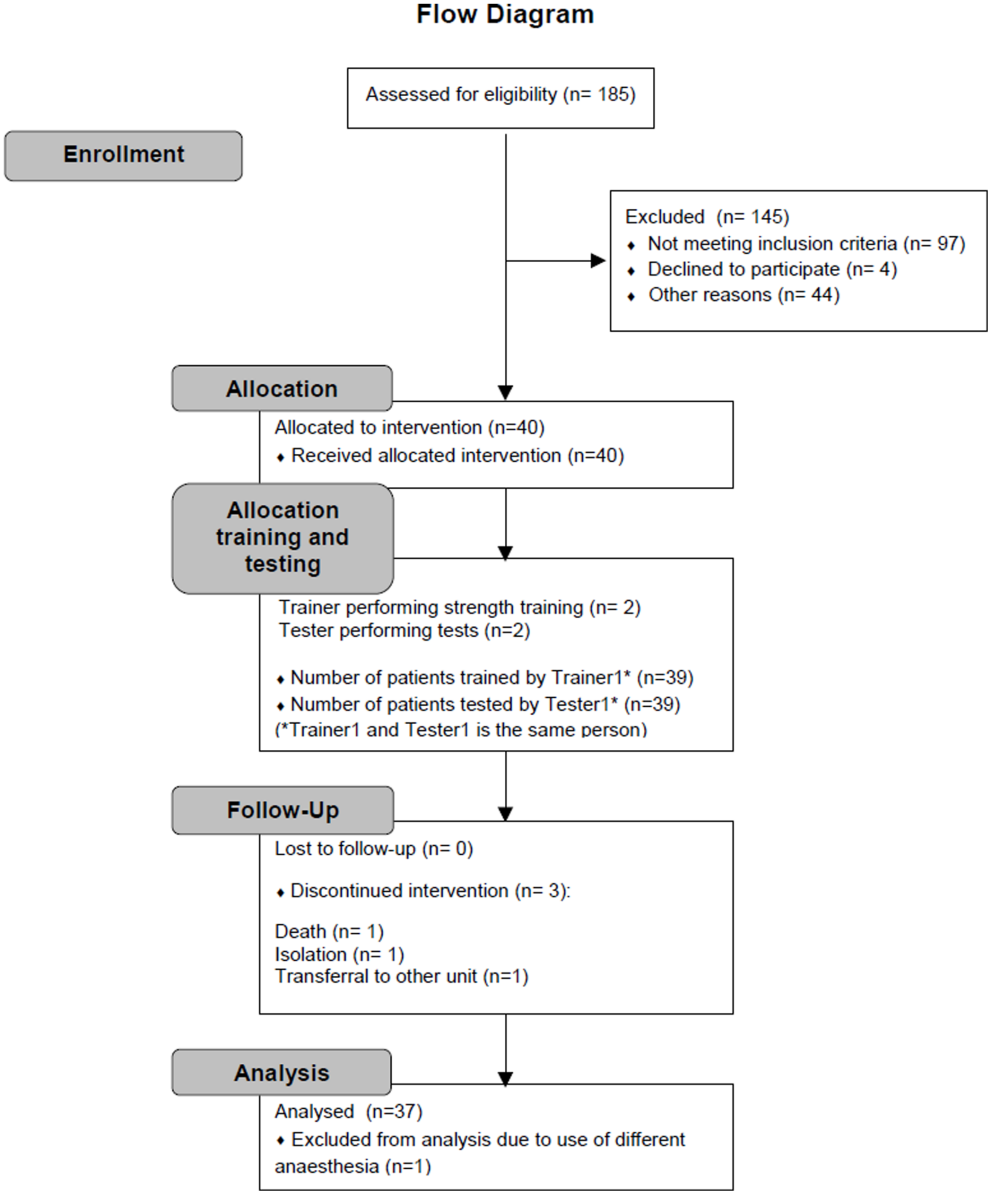
Flowchart of patient enrollment.

A total of 185 consecutive patients admitted with an acute hip fracture between 6^th^ of June 2012 and 6^th^ of December 2012 were assessed for eligibility. The inclusion criteria were: age ≥65 years, ability to speak and understand Danish, able to give written informed consent no later than by the 3^rd^ postoperative day, and home residing with an independent pre-fracture ability to walk indoor, according to a modified and reliable [14], [28] New Mobility Score (NMS) [29] of 2 or more. The exclusion criteria were: postoperative weight bearing restrictions, multiple fractures, postoperative medical complications, terminal illness, neurological impairment, alcoholic addiction and comprehensive co-morbidity. Ninety-seven patients did not meet our inclusion criteria, 4 declined to participate while 44 patients were excluded due to other reasons (Fig. 1). Baseline data for age, sex, body weight, prefracture function (the Functional Recovery Score [30], [31] and the modified NMS [14]), Mental status (Mini Mental State Examination, MMSE) [32], the American Society of Anesthetists (ASA) score [33] and type of fracture were collected from medical records or clinical evaluation of patients.

All patients followed a multimodal fast-track program that included a daily regular physical therapy program (without strength training) at postoperative day 1–3 (weekends included) and 2–5 times per week (weekends not included) hereafter. The regular physical therapy that consisted of 12 specific “hip-related” exercises (repetitions and intensity not standardized) was combined with training of basic mobility activities, such as walking and stair climbing. The program was progressed from actively assisted bed exercises to transfers in and out of bed; sit to stand from a chair, and to more specific and hip-related weight-bearing exercises. Gait reeducation and balance exercises were performed both at the bedside and in a gym located within the hip fracture ward. Walking aids were changed according to the patient’s level of independent mobility. The program did not include progressive strength training of the knee extensors. The number of regular physical therapy sessions and their duration (minutes) were recorded for all patients.

## Intervention

The progressive fractured-limb knee-extension strength training program was conducted once every weekday, and each session consisted of 3 sets of 10 repetitions performed with an intensity of 10 repetition maximum (RM), defined as ±2 RM [34]. Training loads were adjusted on a set-by-set basis and 1-minute pauses separated the sets. The exercise intervention is documented in table 1, according to the study protocol (Please see Protocol S1 and Protocol S2) and descriptors by Toigo & Boutellier [34] and the recommendations from the CONSORT extension for non-pharmacologic treatment [35].

**Table 1:** Strength training descriptors [32].

Variable	Knee-extension exercise
Load, repetition maximum	10
Repetitions per set	10
Sets per session	3
Rest between sets, seconds	120
Sessions per week	5
Contraction modes, seconds	2 Isometric, 3 Eccentric, 3 Concentric
Rest between repetitions, seconds	0
Time under tension, seconds	240
Contraction failure in each set	Yes
Range of motion, degrees	90°
Rest between training sessions, hours	24
Anatomical definition of the exercise (exercise form)	Yes

1. The patient was seated on the bedside, hips and knees in 90° flexion, hands placed on the mattress for support, no contact between the patient’s fractured limb and the floor. The foot of the non-fractured limb resting on a low stool.
2. As a warm-up exercise, the patient performed 5 knee-extensions for each limb separately, without any training loads applied.
3. A weight-cuff matching the patient’s level of 10RM was attached around the patient’s ankle of the fractured limb.
4. The patient’s lower leg was assisted to full knee-extension to allow the first voluntary contraction to be eccentric. The patient held the knee extended for approximately 2 sec before lowering the lower leg into vertical position within 3 sec. and repeated the concentric extension with a duration of 3 sec. (Table 1)
5. The patient kept repeating this exercise for as many repetitions as possible at the required rate until fatigue, defined as not being able to perform a full knee-extension or maintain the rate. The patient was verbally motivated to reach state of fatigue. The exercise was stopped at 15 repetitions without fatigue and the load was increased for the following set.

### Safety Precautions

A senior orthopedic consultant cleared all patients for enrolment in the study, and the intervention was stopped in case of any adverse events related to the strength training or any complications that contraindicated further participation in the study. All potentially adverse events or complications were recorded.

## Outcome Measures

### Training Load

The absolute load (kg) corresponding to 10 RM was recorded for each set at each training session, with the load used in the last of three sets recorded. For each patient, the load used at the last set in the first, middle and last training session was used as data point. Completing less than 2 sets of 10 repetitions in each planned session was defined as a session failure. All causes of session failure were recorded.

### Knee-extension Strength

Maximal isometric knee-extension strength was assessed at a knee joint angle of 90° at the day of inclusion and the day before discharge, using a reliable strap-fixated handheld dynamometer [36]–[38]. Patients, unable to be tested at Day 1 after surgery, were tested the following day, or at the latest at Day 3 post-surgery.

The patients were seated as described for the strength training, with the center of the resistance pad placed 4 cm above the lateral malleolous. The patients performed 1 sub maximal knee-extension followed by 4 maximal knee-extensions for each limb (non-fractured first) with strong and standardized verbal encouragement. The contractions were separated by 1-minute pauses. Maximal isometric knee-extension strength in each leg was subsequently expressed as the maximal voluntary torque per kilo body mass, using the lever arm (distance from the lateral epicondyle of the femur to the center of the resistance pad) and body mass of each patient [25]. The greatest value was identified for each leg and used as data points. These two data points were also used to calculate a strength deficit of the fractured limb as a percentage of the non-fractured limb, and used as the strength deficit data point.

### Hip Fracture-related Pain

Hip fracture-related pain was assessed once before (at rest) and once during all strength training sets and testing sessions, in addition to all functional performance tests using a five-point Verbal Ranking Scale (VRS 0–4 points; 0 = none, 1 = light, 2 = moderate, 3 = severe and 4 = intolerable pain.) The VRS has proved superior to other pain scales [39], and used in previous hip fracture studies [9], [12], [15]. The highest pain level reported from each assessment was used as data points.

### Functional Assessment

During the daily regular physical therapy sessions, the patients were evaluated on their basic mobility capacity by the reliable [40] Cumulated Ambulation Score (CAS) [18], [41]. The post-surgery day of independent ambulation was used as data point [42].

Upon discharge, all patients with an independent walking ability performed the reliable [43] Timed Up and Go test (TUG) [44] 3 times [45] (the fastest of 3 assessments used), and the 10 m fast speed walking test (10 MWT) [46] 1 time, using a rollator as a standardized walking aid in both tests [47]. The 3-step (0–30 s) Tandem test [48] of static balance was recorded as seconds of unsupported stance, while fear of falling was assessed by the Short Falls Efficacy Scale International (FES-I, 7–28 points, high scores indicating fear of falling) [49].

### Statistical Analysis

The most fundamental principle of progressive strength training [50] is to progressively overload the exercising muscle as it becomes stronger [51]. This can be accomplished by progressively increasing the training load (kilograms lifted), while the exercise intensity is kept constant, e.g. 10 RM as in the present study. So, the sample size of the present study was determined based on the primary outcome (change in training loads) and a definition of the smallest clinically relevant increase in training load as being 1.5 kilograms (SD of 2.0) over 5 training sessions. In a fast-track surgery setting, 5 training sessions seems realistic, given an average hospitalization of 12 days, and no training at weekends. To be able to establish this effect, 16 patients needed to be included, using a standard of 80% power and type 1 error rate of 5%. To allow for a fracture type-specific indication of feasibility and an overall dropout rate of 20%, a total of 40 patients were included (20 patients with trochanteric and 20 patients with cervical fractures). We report all analyses related to the feasibility of the intervention – other than that of the training load - using descriptive statistics, and these analyses were considered primary [26]. All analyses related to the secondary outcomes were considered explorative.

All data were examined for normality of distribution (Kolmogorov-Smirnoff and Q-Q plots). Differences between cervical and trochanteric fractures were examined using the chi-square or Fisher exact tests for categorical data, the Students t-test or the Mann Whitney-U test for continuous data, as appropriate, while Paired *t* tests were used to examine changes in maximal isometric knee-extension strength for both limbs, and fractured as % of non-fractured. Repeated measures ANOVAs, in addition to a General Linear Mixed Model (adjusted for fracture type and number of training sessions) with Bonferroni adjustments were used to examine for changes in training loads over time (first, middle and last strength training session). Assumptions for these analyses were evaluated and found valid. Further, the eta-squared statistical analysis for independent-sample t-test (t^2^/(t^2^+N1+N2−2)) was used to examine the effect size of changes in training loads and in the fractured limb maximal isometric knee-extension strength, according to guidelines proposed by Cohen [52]. According to Cohen’s classification, effect size above .01 indicates small effect,.06 moderate effect, and .14 large effect size. Data are presented as means (1 SD) when normally distributed, otherwise as medians (first-third quartile), or as numbers with percentages. The level of significance was set at *P* less than .05. All analyses were conducted with SPSS statistical (version 19; SPSS inc. Chicago, Illinois, USA).

## Results

### Descriptive Characteristics

Characteristics, participation, and outcomes for the 36 patients (18 with a cervical and 18 with a trochanteric hip fracture) who completed the full test and training program between June and December 2012 (9 men and 27 women with a mean age of 79.4 (8.3) years) are presented in table 2 and figure 1. No significant between-fracture type differences in baseline characteristics (Table 2) or in the day of the first or last strength training session existed (Table 3, *P*>.11).

**Table 2:** Characteristics and results of patients following an in-hospital strength training program.

Variable	Total group (n = 36)	Cervical (n = 18)	Trochanteric (n = 18)	*P* Value
Age, mean (SD), years	79.4 (8.3)	78.9 (7.5)	80.4 (9.3)	.75
Men, number (%)	9 (25)	3 (33)	6 (67)	.25
Women, number (%)	27 (75)	15 (56)	12 (44)	
Body weight, mean (SD), kg	65.1 (15.0)	62.6 (16.5)	66.4 (13.3)	.34
NMS, median (IQR), 0–9 score	9 (4.5–9)	9 (5–9)	9 (4.5–9)	.66
ASA, median (n = 1/2/3/4 score)	2 (8/19/9/0)	2 (4/9/5/0)	2 (4/10/4/0)	.81
Hindsoe, median (IQR), 0–9 score	9 (8–9)	9 (7.75–9)	9 (7.75–9)	.68
FRS, median (IQR), 0–100 points	97 (72–100)	94.5 (71.25–100)	97 (70.25–99)	.87
MMSE, median (IQR), 0–30 points	26 (4.4)	25.56 (5.1)	26.4 (3.6)	.58
**Knee-extension strength outcomes**				
MVT f % nf at training start, mean (SD)	50.3 (33.6)	61.5 (40.0)	38.9 (21.4)	.04
MVT f % nf at discharge, mean (SD)	68.2 (25.2)	80.0 (21.8)	56.5 (23.3)	.004
MVT nf at start, mean (SD), Nm/kg	0.87 (0.4)	0.79 (0.4)	0.95 (0.4)	.19
MVT f at start, mean (SD), Nm/kg	0.37 (0.2)	0.41 (0.2)	0.33 (0.2)	.19
MVT nf at discharge, mean (SD), Nm/kg	0.95 (0.4)	0.87 (0.3)	1.03 (0.5)	.22
MVT f at discharge, mean (SD), Nm/kg	0.61 (0.3)	0.69 (0.3)	0.54 (0.3)	.14
**Functional outcomes**				
Independent in basis mobility (CAS = 6), number (%)	29 (81)	16 (55)	13 (45)	.21
Day of independence in basic mobility, mean (SD)	6.2 (2.3)	6.1 (3.0)	6.4 (2.0)	.73
10 MWT, m/s, mean (SD), (n = 28^a^)	0.59 (0.3)	0.61 (0.3), (n = 14)	0.57 (0.3), (n = 14)	.79
TUG, s, mean (SD), (n = 27^a^)	30.9 (20.3)	29.5 (18.6), (n = 14)	32.4 (22.7), (n = 13)	.72
Tandem, s, mean (SD), (0–30 s), (n = 29^b^)	17.7 (10.2)	19.1 (10.5), (n = 15)	16.3 (10.1), (n = 14)	.47
Short FES-I (7–28 points), mean (SD), (n = 32^c^)	15.9 (10.2)	14.1 (5.1), (n = 15)	17.5 (7.6), (n = 17)	.14

Abbreviations: NMS; New Mobility Score. ASA; American Society of Anaesthesiologists physical classification system. Hindsoe Test: Test of memory. FRS: Functional Recovery Score. MMSE: Mini Mental State Examination. Post-opr.: Post-operative. MVT; maximal voluntary torque. f: fractured limb. nf: non-fractured limb. CAS: Cumulated Ambulation Score. 10 MWT: 10 m fast speed walking test, TUG: Timed up and go test using standardized aid (rollator), Tandem: Tandem test of balance. Short FES-I: Falls Efficacy Scale –International.

a Functional tests were only performed in patients with an independent walking ability with a rollator at discharge.

b Balance test was only performed in patients able to stand without support.

c Four missing tests due to lack of time since discharge test was performed on the actual day of discharge (n = 3) or delirium (n = 1).

**Table 3:** Program timeline, Adherence to programme and detailed physical therapy applied at unit according to regular regime.

Variable	Total group	Cervical	Trochanteric	*P* Value
	(n = 36)	(n = 18)	(n = 18)	
Strength training				
First session day, mean (SD), Post-opr.	2.4 (0.7)	2.4 (0.7)	2.4 (0.7)	.81
Last session day, mean (SD), Post-opr.	8.6 (4.2)	7.4 (4.2)	9.7 (4.1)	.11
Day of discharge, mean (SD), Post-opr.	12.3 (6.6)	10.0 (4.6)	14.5 (7.5)	.04
Possible sessions, mean (SD)	5.1 (2.6)	4.2 (2.5)	6.0 (2.4)	.03
Training sessions conducted, mean (SD)	4.3 (2.2)	3.6 (2.0)	4.9 (2.2)	.06
Adherence to strength program				
No. (%) of possible training sessions	212	86 (41)	126 (59)	.07
No. (%) of training sessions conducted	183 (86)	75 (41)	108 (59)	.03
No. (%) of patients missing sessions	20 (56)	8 (40)	12 (60)	.18
No. (%) of strength sessions failure due to				
Exhaustion	16 (55)	6 (38)	10 (62)	.01
Nausea	7 (24)	4 (57)	3 (43)	.66
Hip fracture-related pain	3 (10)	0	3 (100)	
Logistics	2 (7)	0	2 (100)	
Cognitive dysfunction	1 (4)	1 (100)	0	
Regular physiotherapy without strength training				
Sessions, median (IQR), days	6 (4–7)	5 (3–6.25)	6 (6–7.5)	.02
Total time, mean (SD), min	126 (56)	109 (47)	144 (61)	.07
Time per session, mean (SD), min	22 (7)	21 (7)	22 (7)	.56
Total functional therapy time, mean (SD), min	74 (47)	66 (45)	84 (47)	.19
Total exercise therapy time, mean (SD), min	52 (30)	43 (28)	60 (31)	.18

### Training Loads

**Figure 2:**
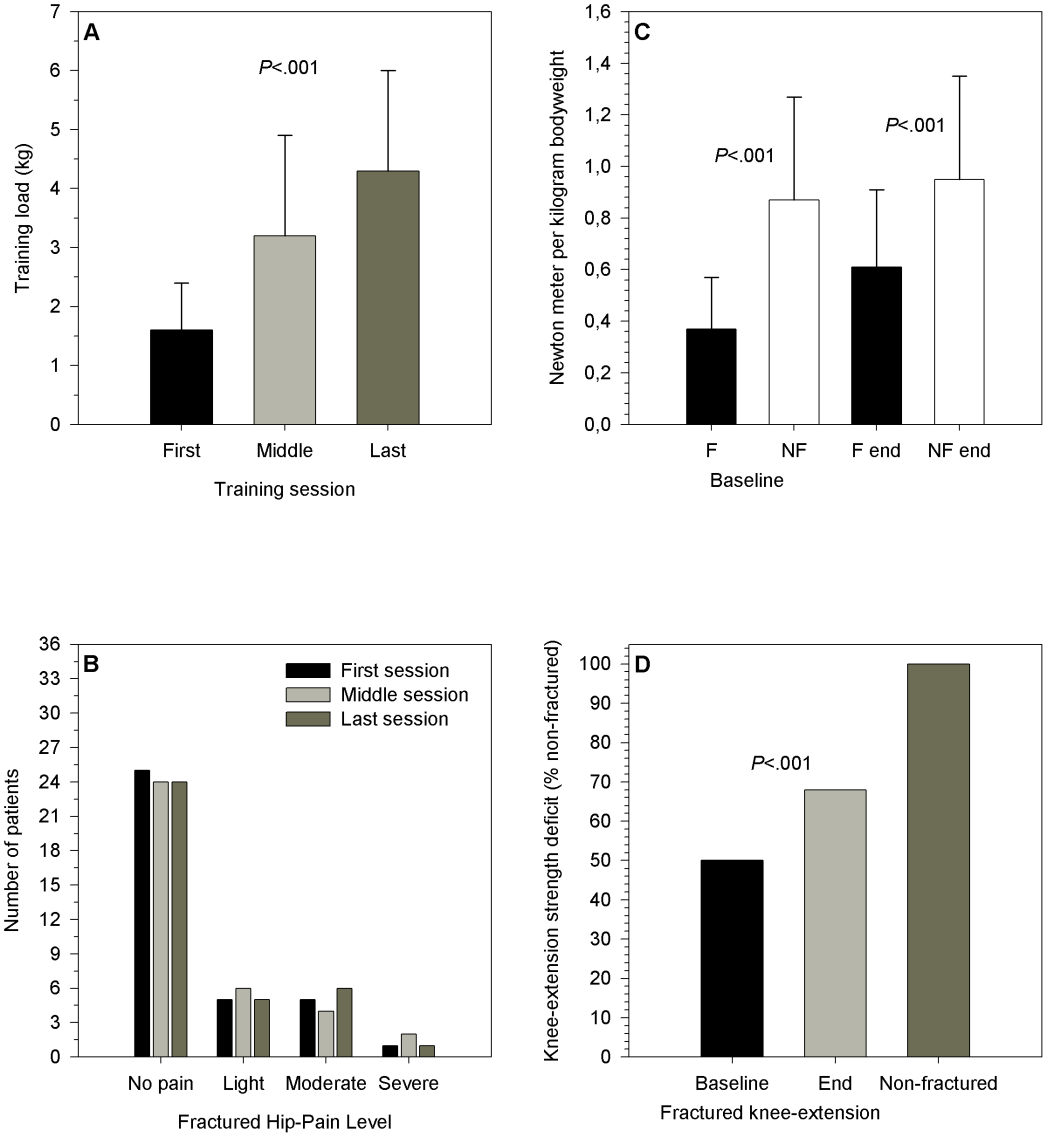
Outcomes on training load, hip-pain and knee-extension strength. A) Progression of training loads (kg) during the first, middle and last training session. B) Hip fracture-related pain during the first, middle and last strength training session. C) Knee-extension strength (Nm/kg), fractured (F) and non-fractured (NF) limb at baseline and discharge (end). D) Fractured limb knee-extension strength (% non-fractured) before and after training.

The absolute loads (the kilograms that were lifted) increased progressively from a mean of 1.6 (0.8) to 4.3 (1.7) kilograms (*t* (48.1) = −8.66, *P*<.001, eta squared = .52) (Fig. 2A), over a mean of 4.3 (2.2) training sessions. The General Mixed Model showed no significant interaction between type of fracture and progression of training loads over time, (*P* = .121, partial eta squared = .12), while there was a substantial progression in training loads for both fracture types (within-subjects analysis, *P*<.001, partial eta squared = .38). The main effect between fracture type groups comparing progression of training loads was not significant, (between-subjects analysis, *P* = .862, partial eta squared = .001).

### Adherence to Program

The initial strength test was postponed to Day 2 in 11 patients due to nausea (n = 4), tiredness (n = 4), pain (n = 2) and delirium (n = 1) and to Day 3 in 1 patient due to tiredness. Strength training was initiated at a mean of 2.4 (0.7) days post-surgery and ended at day 8.6 (4.2) (Day 0 = day of surgery) (Table 3). One hundred-eighty three (86%) out of 212 possible training sessions were completed, and 16 patients completed all planned sessions with no adverse events related to the strength training. Reasons for session failure were mainly related to exhaustion (55%) and nausea (24%), while only 3 sessions (2 patients with a trochanteric fracture) of a total of 212 sessions were not started or completed, due to a VRS pain score of 3 (severe pain) (Table 3).

#### Hip fracture related pain

More than 80% of the patients reported no or light pain during strength training and with the same number of patients reporting moderate or severe hip pain during the first (6 patients) and last (1 patient) training session (Fig. 2B). Similarly, more than 85% (n = 31) reported no or light pain during the baseline test of knee-extension strength. Upon discharge, all patients completing the program (n = 36) reported no or light pain at rest, while 32 (89%) patients reported no or light pain during the strength test. No patients reported intolerable pain (VRS = 4) during any of the above-mentioned sessions or outcome assessments.

#### Strength deficit

The fractured limb knee-extension strength increased significantly from a mean of .37 (0.2) to .61 (0.8) Nm/Kg (*t* (70) = −3.97, *P*<.001, eta squared = .18, Fig. 2C), while deficits decreased from an average of 50% (34) at baseline to 32% (26) at discharge (*P*<.001, Fig. 2D), and was more pronounced at discharge in patients with a trochanteric fracture (43%), compared to those with a cervical fracture (20%, *P* = .004, Table 2).

### Functional Outcomes

No significant between fracture-type differences were found for the 10 MWT, TUG and Tandem test or in scores of the short FES-I questionnaire upon discharge from hospital (Table 2, *P*>.14).

## Discussion

The main finding of the present study was that physical therapy including progressive strength training (10 RM) implemented in the acute ward immediately after hip fracture surgery seems feasible. That is, we found that the training load increased substantially, and more than that defined as the smallest effect worth detecting, while hip fracture-related pain was low to moderate and adherence high. The results also support that this type of physical therapy exercise might establish efficacy in reducing the knee-extension strength deficits of the fractured limb.

### Training Load

The absolute training loads of the fractured limb increased progressively by an average of 188% from the first to the last training session, demonstrating a large observed effect size.

This underlines the importance of adjusting training loads on a set-by-set basis, when implementing this exercise modality clinically. Our increase in training load corresponds well to that reported in a fast-track total knee arthroplasty program with immediate progressive strength training of the knee extensors [25]. In the current study, the increase in training load was achieved with high adherence as reflected in less than one training session failure per patient, and with the 4 (10%) dropouts unrelated to the strength training or other assessments.

### Hip Fracture-related Pain

Pain during the knee-extension strength training was generally low, even though we used the highest pain value recorded in each session as data points. Similarly, pain did not appear to influence the muscle strength testing, in accordance with that previously reported [25]. This is, however, in contrast to previous reports of hip fracture-related pain influencing in-hospital functional performances, such as walking with weight bearing on the operated leg, shortly after hip fracture surgery [12], [15], and lower than that previously reported by Sherrington et al. at a later time-point [11]. The reason may be that both the test of muscle strength and training were performed using an open kinetic chain, where the foot is not fixed in space, as opposed to a closed kinetic chain exercise or task, where the foot is fixed in space (e.g. rise from a chair). So, even though the quadriceps muscle is loaded heavily during the strength training exercise, little compressive or shear forces are likely to occur in the operated hip, as opposed to those occurring during rising from a chair, where substantial hip extensor power is generated [53]. Another explanation may be that the multimodal analgesia regimen consisting of postoperative continuous epidural analgesia (96 hours postoperatively), paracetamol, NSAID and supplied by opioids if needed [54], may differ from that used in e.g. the study by Sherrington et al [11].

### Strength Deficit

The fractured limb knee-extension strength deficit decreased with approximately 18% when calculated as a mean of the 2 fracture type groups. However, greater deficits were seen for patients with a trochanteric as compared to those with a cervical fracture, and neither group reached the level of the non-fractured limb. With respect to discharge status, the deficit was reduced to 32% in the present study, as compared to 53% in a previous study, in which patients followed the same multimodal fast-track program, but did not undergo strength training [9]. This suggests that this type of exercise implemented immediately following surgery may be effective in reducing the strength deficit of the fractured limb, as a large effect size for changes in maximal isometric knee-extension strength was found, but needs experimental verification in a randomized controlled study design. Supporting a likely effect, Mitchell et al [10] found good compliance with a 6-week progressive quadriceps strength training program implemented in an inpatient rehabilitation setting shortly (median of 15 days post-surgery) after hip fracture. These authors found that the fractured leg extensor power increased significantly by 157% in the strength training group, compared to 63% in the control group at the 6-week follow up [10]. This was obtained although the exercise intensity was started at 50% of 1 RM (equal to >15 RM) and first increased to 80% of 1 RM (equal to 7 RM) in the last two of the six weeks of training, as opposed to an intensity of 10 RM (equal to 75% of 1 RM) from day 1 in the present study. These findings are supported by a newly published 6-week progressive strength training program (twice weekly, and training loads adjusted on a set-to-set basis) by Overgaard and Kristensen [22], conducted in an outpatient municipality setting. The later study included home-dwelling patients at a mean of 17.5 (5.7) days after hip fracture surgery, and reported an increase in fractured limb knee extension strength by 67%, while the fractured limb strength deficit (% non-fractured) was reduced from 40% to 17% at end of the program.

To that, a randomized controlled study by Binder et al [21] with a strength training intervention started after ceased standard physical therapy (average of 14 weeks post hip fracture surgery) in community dwelling elderly participants, reported effect of the intervention. Further, Sherrington et al [11] reported in a 2-week inpatient rehabilitation program, that both patients who followed a weight-bearing and a non-weight-bearing exercise program improved markedly on strength and functional performances.

Finally, the same overall effect in favor of strength training after hip fracture was found by Singh et al [55], who implemented a 1-year community-based strength training program 6–8 weeks after the fracture and found a positive effect on ADL dependency, reduced nursing home admissions and mortality, when compared to usual care. On the contrary, a yearlong low-intensity home exercise intervention program showed no significant between-group differences for functional performances [56].

Nonetheless, although an increasing number of studies provide evidence for strength training as an important intervention after hip fracture, none has so far succeeded in eliminating the fracture limb strength deficit, and none has started the intervention in the acute ward and exercised daily as in the present study. Hence, a care trajectory with progressive strength training implemented in the acute ward shortly following surgery, and continued on an outpatient basis after discharge is indicated to be effective towards several outcomes.

### Functional Outcomes

No significant difference between fracture type groups was found in any of the functional performances, which is in contrast to that previously reported, at the same time point after hip fracture surgery in patients who did not undergo strength training [42], [57]. The explanation hereto is probably lack of power, as the present study was not powered to investigate this specifically. Mitchell et al [9] reported a mean gait speed of 0.38 m/s 10 weeks after intervention in a comparable population, which is considerably lower than the 0.59 m/s found in this study at discharge from the acute ward. Compared to cut-points for severe mobility limitation, a gait speed of 0.59 m/s is still way below the cut-point of 1.22 m/s measured in well-functioning older adults [58], and should be addressed in rehabilitation after a hip fracture. Nonetheless, according to a recent systematic review [59], most of the patients included in the present study will be able to walk outside their house after discharge.

### Study Weaknesses and Strengths

The included patients had a relatively high cognitive and prefracture functional level, which restricts our findings to patients with similar characteristics. Still, this is in accordance with previous studies [8]–[10]; [14], and the intervention is considered feasible within the study population. In general, the same physical therapist supervised all training and testing, which might have influenced results. However, we kept baseline data inaccessible to the tester until the study was completed, to minimize this influence. Finally, the promising results could to some extent be argued related to the regular physical therapy applied in the ward or spontaneous recovery. The greatest strength of our study is the well-described and simple intervention, making it realistic and low in cost to implement as a part of routine practice in the acute ward.

## Conclusion

Physical therapy including progressive strength training (10 RM) implemented in the acute ward immediately after hip fracture surgery seems feasible. That is, we found that the training load increased substantially, and more than that defined as the smallest clinically relevant increase, while hip fracture-related pain was low to moderate and adherence high. The findings suggest that progressive strength training may reduce the knee-extension strength deficits of the fractured limb, but the intervention and the clinical efficacy need confirmation in a randomized controlled design.

## Supporting Information

Protocol S1 **Complete study trial protocol as approved by ethics committee, English version.**
(DOC)

Protocol S2 **Complete study trial protocol as approved by ethics committee, original language.**
(DOCX)

Checklist S1 **STROBE checklist of items that should be included in reports of cohort studies.**
(PDF)
