# Supplementary material for: Feasibility of Progressive Strength Training Implemented in the Acute Ward after Hip Fracture Surgery
Source: PLoS One. 2014 Apr 3;9(4):e93332. doi: 10.1371/journal.pone.0093332 (PMC3974729; doi:10.1371/journal.pone.0093332)
Supplement: Protocol S1 — Complete study trial protocol as approved by ethics committee, English version. (DOC) [file pone.0093332.s001.doc]

Physiotherapy including progressive resistance training started early after surgery for hip fracture: Can this be done and what is the effect?

**Investigator and Project Manager**Morten Tange Kristensen, Senior Researcher, PhD, Department of Physiotherapy 236 and Department of Orthopaedic Surgery 333, RegionH, Hvidovre Hospital, tel +45 38 62 61 91
E-mail: morten.tange.kristensen@regionh.dk

**Clinical responsible**Henrik Palm, MD, Department of Orthopedic Surgery 333, RegionH, Hvidovre Hospital,
E-mail: hpalm@dadlnet.dk

**Other investigators**Henrik Kehlet, Professor, PhD, Section of Surgical Pathophysiology 4074, RegionH, Rigshospitalet.
Thomas Bandholm, Senior Researcher, PhD, Clinical Research Unit 136, Department of Orthopedics and Physiotherapy 236, RegionH, Hvidovre Hospital.

**Study location**
Hvidovre Hospital, Kettegård All 30, 2650 Hvidovre, tel +45 36 32 36 32

PROTOCOL SUMMARY………………………………………………………………………………………………….. 3 PURPOSE OF THE STUDY……………………………………………………………………………………………….. 4
*Primary purpose*..……………………………………………………………………………………………………………4
BACKGROUND…………………………………………………………………………………………………………….4
STUDY DESIGN……………………………………………………………………………………………………………5
STUDY TYPE……………………………………………………………………………………………………………….5
*Randomization*……………………………………………………………………………………………………………….5
*Blinding*…………………………………………………………………………………………………………………...…5
*Schedule*…………………………………………………………………………………………….………………………..5
*Interruption*…………………………………………………………………………………………………………………..5
*Data recording*………………………………………...……………………………………………………………………..6
PATIENT POPULATION…………………………………………………………………………………………………...6
*Inclusion criteria*……………………………………………………………………………………………………………..6
*Exclusion criteria*…………………………………………………………………………………………………………….6
PROCEDURES………………………………………………………………………………………………………...……6
STANDARD OF TRAINING during hospitalization…………………………………………………………………….....6
*Physiotherapy including strength training*…...………………………………………………………………………...……6
*Physiotherapy without strength*……………………………………………………………………………………………...7
AFTER PRINTING………………………………………………………………………………………………………….7
REGISTRATIONS……………………………………………………………………………………………………..……7
*Primary outcome*………………………………………………………………………………………………………..……7
*Secondary endpoints*…………………………………………………………………………………………………………7
*Other parameters*………...………………………………………………………………………………………………..…8
SAFETY………………………………………………………………………………………………………………….….8
*Safety*………………………………………………………………………………………………………………………...8
*Education*……………………………………………………………………………………………………………….……8
ETHICS……………………………………………………………………………………………………………………...9
OBTAINING CONSENT…………………………………………………………………………………………………....9
DATA PROCESSING AND DATA ANALYSIS…………………………………………………………………………..9
ECONOMY………………………………………………………………………………………………………………...10
PUBLICATIONS…………………………………………………………………………………………………………...10
*Layman's summary*…………………………………………………………………………………………………………12
PATIENT INFORMATION - PURPOSE AND COURSE OF THE INVESTIGATION………….……………………...13
PATIENT INFORMATION - GENERAL ETHICAL ASPECTS AND STATUTORY REQUIREMENTS…………….15
TEST PERSON 'S RIGHTS IN BIOMEDICAL RESEARCH (attached THE WRITTEN PATIENT INFORMATION)..16

**Protocol Summary**
Elderly patients with a hip fracture are a particularly vulnerable group with high morbidity and mortality (1-3) and some patients do not regain previous level of physical function (4, 5). The most recent Cochrane review that assesses the effect of physical therapy concludes evidence from randomized trials insufficient to determine the effectiveness of different types of exercise used in the rehabilitation after hip-fracture surgery (6).

Patients with a hip fracture admitted to Hvidovre Hospital have since September 2002 been treated in the fast track concept (7, 8) in a specialized unit at the Department of Orthopedic Surgery. The treatment is optimized and intensified regarding surgery, nutrition, pain management, physical therapy, etc. during hospitalization. Despite the optimized efforts, patients who are discharged directly to their own home after following a standard course are admitted in average 16 (SD 14) days and still some patients are discharged to 24hour rehabilitation units in the primary sector. A study from the unit has shown that 32 % of patients fall again within 6 months after discharge (9). Other data collected in the unit have shown that quadriceps muscle strength of the operated limb among patients discharged directly to their own home is less than 50% of the strength of the healthy limb (10), despite the fact that they have followed an optimized treatment course.

Despite no clear evidence, that strength training is long term effective for patients with a hip fracture (6), it is still considered an essential component of the rehabilitation process. It is well known that resistance training increases muscle strength in healthy elderly people (11) and patients with a hip fracture, even when initiated 16 days after surgery at the earliest (12). This is also supported by two other studies where strength training was started at an even later time-point after discharge (13, 14). All three studies showed effects of strength training at the end of the intervention compared with the control group, but either there were too few participants or too short follow-up time to conclude on the effects of strength training as an intervention, as well as the intervention was implemented so late that the loss of muscle function already was established. Subsequently, since the elderly experience an average decrease in muscle strength of 15% after 10 days of bed rest (15, 16) and already has low muscle strength due to old age, early strength training in patients with hip fractures seems rational to avoid a major loss of strength and physical function. For example, a study among elective patients operated with total hip arthroplasty showed effect of strength training started immediately after surgery (17). It is unknown whether elderly patients with a hip fracture can participate in physical therapy including strength training started immediately after surgery and whether the effect is greater than physical therapy without strength training.
We therefore wish to perform *(1)* a study of the feasibility of strength training started the day after surgery, and *(2)* a randomized study with implementation of strength training the day after surgery, where 100 elderly patients with hip fractures admitted in the Hip fracture unit at Hvidovre Hospital are randomized to physical therapy with or without progressive resistance training.
The study is part of the "Hip fracture project", Department of Orthopedic Surgery, Hvidovre Hospital, and with an expected duration of 30 months. It should be emphasized that the investigation is not supported by or associated with the pharmaceutical industry, and none of the participants have financial or other interest in the study.
 

**Purpose of the study**Primary purpose:
To examine *(1)* the feasibility of strength training in the early postoperative phase after hip fracture, *(2)* the effects of physical therapy with and without progressive resistance training in the early postoperative phase after hip fracture.

**Background**Patients with a hip fracture are a particularly frail group with high morbidity and mortality (1-3) and many patients do not regain previous level of physical function (4, 5).
The fast track multimodal treatment program for patients with a hip fracture (7), which all patients at Hvidovre Hospital follows, focuses on early surgery, prevention of per operative hypo ischemia, optimized per operative analgesia, adequate nutrition and fluid therapy and early mobilization and physical therapy. In this regimen, patients are mobilized and receive physical therapy 7 days a week (first 3 days after surgery, then weekdays only).

The department has developed a new scoring system, the Cumulated Ambulation Score (CAS) (18,19) describing the level of basic mobility, defined as the ability to get in and out of bed, sit-to-stand chair raise and walk with a suitable walking aid. The CAS score for the first 3 days after surgery for patients admitted from their own homes, has been associated with; discharge within 14 days, discharge back to own home, 30-day mortality and medical complications. A six-month follow-up study (5) from the same unit, have shown that even among the best of the patients, 70% still had to use a walking aid compared to only 40 % before fracture, and that 32% had experienced one or more fall episodes after discharge (9). The same study also showed that it was predominantly patients with a New Mobility Score (0-9) (20) before hip fracture of ≥ 6 (high level), whom it was possible to follow.
Patients are characterized by a high average age, which for many patients are associated with sarcopenia (age-related loss of muscle mass, strength and physical function) (21) and a deterioration of this is a known result of a hip fracture. Data collected in the unit have shown that, despite this intensive rehabilitation program, the muscle strength in the operated limb among patients being discharged directly to the own home, represents less than 50% of the strength in the healthy limb (10), similar to levels seen in a previous study (22). In addition, the strength loss was significantly greater among patients with a trochanteric hip fracture, compared to patients with a cervical hip fracture and the degree of strength loss was strongly associated with the degree of swelling (edema) in the operated limb (10). The importance of fracture type is further demonstrated in a study showing that the probability of achieving independence in basic mobility assessed by the CAS and to return to the original housing is less for patients with a trochanteric fracture compared to patients with a cervical fracture (23). Strength training increases muscle strength in healthy elderly persons (11) and three studies have shown short-term effects of resistance training in patients with a hip fracture initiated at a later stage of the rehabilitation program (12-14) as well as in elective patients operated with total hip arthroplasty and starting strength training immediately after surgery (17). It is therefore considered rational to evaluate physical therapy including strength training, starting in the very early postoperative period in patients with a hip fracture.

**Study Design**
**Study type**
Part one: The study of the feasibility of strength training during hospitalization is planned conducted with 40 patients meeting the inclusion criteria for the randomized study. This project will be completed prior to the initiation of the randomized trial.

The randomized part of the study is conducted as a single-blind study:
 Randomization

Strength

Run in

Treatment period Follow up

Control

Test

Screening

Test

Time ____________________________________________________________________________________

Opr. 1^st^ post opr. day 10^th^ post opr. day/discharge 16 weeks

**Randomization**
Patients are randomized to one of two treatment groups. A computer compiles randomization list. Based on randomization, ​​envelopes are made with patient number, including allocation of patients to standard physiotherapy + strength training vs. standard physiotherapy without strength training as it is today with exercises and functional training. The envelopes are opened after inclusion for the individual patient only. The randomization list is kept in a sealed envelope by the clinically responsible. Patients are stratified by type of fracture, so that each group includes an equal number of cervical versus inter-/subtrochanteric fracture.

**Blinding**Randomized study: Patients, doctors, nurses and involved physical therapists are for obvious reasons not blinded, but test at inclusion, discharge, and 16 weeks will be carried out by blinded personnel without knowledge of treatment group.

**Timetable**Part one: The 40 patients are planned for inclusion in the period mid 2012 to ultimo 2012. This part of the study found strength training feasible and without pain or other adverse events associated.
Randomized part: Recruiting of the patients is expected to begin mid 2013 and the study is expected to extend over a period of approximately 18 months, provided the expected inclusion of 6 patients per month.

**Interruption**
The individual patient may withdraw from the study at any time. Patients may also be excluded from the study at any time based on the investigator's evaluation.

**Data registration**
All data relevant for the study are recorded in a specific ​​form. Data of each enrolled patient are listed in a personal form.

**Population**
Randomized part: Calculations have shown that in order to show a 30% reduction in the difference in strength between the operated and the healthy limb, with a significance level of 0.05 and 80% strength, 38 patients needs to be included in each group. Due to the expected dropout rate, 100 elderly patients admitted from their own homes to the acute ward with a hip fracture will be included.

**Inclusion criteria:**Patients aged ≥ 60 years admitted to the acute ward with a diagnosed hip fracture (cervical, pertrochanteric or subtrochanteric fracture) and
· Able to speak and understand the Danish language
· Able to give informed consent
· Home-dwelling with an independent physical function estimated as a New Mobility Score of ≥2 indoor.
**Exclusion Criteria:**
· Multiple fractures requiring surgery
· Postoperative surgical mobilization restriction
· Patient unwilling to participate in appropriate rehabilitation
· Terminal illness

· Patients who want an observer present at the information interview, but where such one is not available.

**Procedures
Part One:** Strength training is an optional treatment in the regular physical therapy treatment today, and will be attempted systematically implemented starting day 1 or 2 postoperatively. The aim is strength training conducted every weekday during in-hospital stay.

**Randomized part:**1. Information and provision of informed consent before or at the latest, on the 2nd postoperative day after admission.
2. Randomization to two groups of 50 patients performed by the project manager or one of the other investigators.

**Standard for physical therapy during hospitalization**

**Physical therapy including strength training**
Daily on weekdays (first 3 days after surgery including weekends) during hospitalization with a physical therapist focusing on independence in basic activities (same program as the control group, physical therapy without strength training) but also including:
Operated leg strength trained with knee-extension exercises 0-90 degrees. The starting position is sitting in the chair, on the bed or couch with 90-degree flexion of the knee. The concentric and eccentric movement is performed slowly and controlled and performed with ankle weights.
The strength training is conducted as follows from 1st postoperative day during hospitalization:
3 sets of 10 repetitions, 10 RM (repetition maximum) load.
Patients unable to complete the entire program on a given day, will perform as many sets and repetitions as possible.

**Physical therapy without strength training**
Exercising "ankle flexions" as bed exercises and hereafter daily exercise of: getting out of/in to the bed, chair raise/sit in chair, walking with suitable walking aid and climbing stairs during in-hospital stay.

**After discharge**
All patients follow standard rehabilitation course in the primary sector (usually up to 8 class exercises).

**Registrations**

# Primary outcomes

**Physiotherapy and Mobilization**
- **During in-hospital stay and at discharge:**
The New Mobility Score (NMS) (20, 24) is used for the assessment of pre-operative physical function. Patients are assessed at a daily basis on their ability to perform basic mobility tasks, defined as: getting out and into the bed, sit to stand from a chair and walking with relevant walking aid. The Cumulated Ambulation Score (18) is used to describe this level of basic mobility. Furthermore, the time recorded in the Timed Up and Go test (TUG) (25-27), and 10 meters walk (fast speed walking), which assesses functional mobility are registered upon discharge. The tests are part of the regular physical therapy conducted in the acute ward. In the event of a patient being unable to perform strength training or a given physical function independently, the limiting factors that apply are registered. The circumference of thigh and lower leg and – both fractured and healthy limb is measured at inclusion and discharge as in previous study (10).
Isometric strength is measured with a handheld dynamometer as early as possible after inclusion.
All re-tests are performed on the 10th postoperative day if possible and/or the day before discharge.

**- 16. week post operatively follow up**

TUG, 10 Meter Walking test, NMS, isometric strength both limbs.

**Secondary outcomes**
**Admission time and fall episodes**Admission time is recorded as the time from arrival at hospital to the point of time when the patient is either discharged or has died. The post-operatively day of patients meeting the discharge criteria are registered, as well as the day of prescription for a 24hour-rehabilitation unit or nursing home facilities for patients who can not be discharges to their original housing.

Training sessions during in-hospital stay and outpatient rehabilitation are recorded for both groups, including number of visits and type of training.

**Other parameters**Bodyweight and height

**Safety**The safety issue is related to strength training. Studies including strength training have not previously described safety problems, and the program planned for this study does allow an adaptation phase. Thus, based on the available evidence the patients are not exposed to any additional safety risk, other than that of possible slight muscle soreness after the first strength training sessions. The study is not blinded to the physical therapists, why the identification of possible safety issues will be easy.

**Education**
Observations and measurements are carried out by physical therapists, doctors and nurses associated with the project.

**Adverse events**
In this context adverse events are defined as any unintended, unfavorable finding, symptom or disease that can be connected to the strength training, whether it is deemed to have a correlation with this or not. Adverse events are recorded by spontaneous registration as well as by open questioning.

**Serious adverse events**
In this context serious adverse events are defined as events or reactions that cause:
· Death
· Life-threatening situations
· Admission to hospital or prolongation of existing hospitalization
· Permanent or severe disability/incapacity
Serious adverse events must be evaluated by the investigator for possible correlations with treatment in the intervention group, to consider whether there is a reasonable possibility that the adverse event has been caused by that. The following factors are included in the evaluation:
- Consistency of time.
- Conformity with known effects of the treatment.
- Alternative reasons.
If a serious adverse event is considered to have a causal correlation with the treatment, the investigator must consider the study to be terminated ahead of time.

**Ethics**
No studies were found, dealing with intensive strength training implemented in the very early postoperative period and its importance to the rehabilitation of patients with hip fracture, why this study is so absolutely relevant, as well as it as earlier described, should not pose any safety risk to the patient. The study will be conducted in accordance with the principles of the Helsinki Declaration.
Patients are covered by Hvidovre Hospital patient insurance.
The protocol including patient information and consent forms for the randomized study is approved by the Research Ethics Committee of the Capital Region (HA-2007-0127) and registered with ClinicalTrials.gov.
The investigator is responsible for informing the Research Ethics Committee of any serious adverse event and/or major changes in the protocol. The coordinating investigator files all correspondence.

**Obtain consent**
All patients considered eligible receive oral and written information about the study. Inclusion takes place after obtaining informed consent from the patient himself. It is the responsibility of the investigator to provide patients with comprehensive written and oral information on the progress, purpose, risks and potential benefits of the study.
The project manager contacts the patient in person after admission to the Department of Orthopedic Surgery 312, Hvidovre Hospital. At recruitment, the patients should be informed that it is a scientific study, that the patient has the right to have an observer present during the information and that the patient may request a certain period for consideration after receiving the study information. To ensure that patients have at least 24 hours of consideration before signing of informed consent, the written and oral information is given as soon as possible after surgery. This, in order to provide the intervention group the possibility of beginning the strength training on the day after surgery. The oral information is provided in connection with the written information and will follow the content of this with further explanation given as needed. The oral information is adjusted according the patient's age, social affairs and education. It is ensured that the patient has understood the contents of both the oral and the written information. The project manager or another authorized person in the research group provides the oral information. The project manager shall ensure that such authorized person is thoroughly informed about the project and professionally qualified to provide the information. Consent to participate in the study is sought for on the day after providing information and after the patient has had a minimum of 24 hours for consideration on participation.

**Data processing and data analysis**

An assigned project number identifies all enrolled patients. At end of study, all personal identifiable data will be destroyed. Patients are informed orally and written, that data is stored and analyzed in a computer, the patient's anonymity will be ensured, and that the study complies with law of data protection. Assuming normally distributed data, the primary analysis will be repeated measures analysis of variance (ANOVA) to determine the systematic difference between the intervention and control group. Furthermore an intention-to-treat analysis will be carried out. The Data Inspectorate has approved the study J.no. 2007-41-1573.

**Financial disclosures**The study is part of the Hvidovre Hospital "Hip fracture project". It is emphasized that the study is not supported by the pharmaceutical industry or by any other institutions with financial interest in the studied problem, and that no participants in the project have financial or other interests in the studied problem.
Financial assistance for completion of the study will be applied for, but none of the potential sponsors in the project will have any influence or financial disclosure in the organization, implementation and interpretation of the data collected in the project.
 **Publication**The study results, both positive and negative, are planned for publication in an international English journal. The project manager prepares the manuscript, is corresponding author and is listed as the first author. All investigators are listed as authors.
Similarly, the results will be presented at national and international congresses. Provided positive experience with strength training for patients with a hip fracture, the procedure for this will be sought incorporated into national guidelines.

**Reference List**

1. Rasmussen S, Kristensen BB, Foldager S, Myhrmann L, Kehlet H. [Accelerated recovery

program after hip fracture surgery]. Ugeskr Laeger. 2002; 165:29-33

2. Sharrock NE. Fractured femur in the elderly: intensive perioperative care is warranted. Br J

Anaesth. 2000; 84:139-40

3. Foss NB, Kehlet H. Mortality analysis in hip fracture patients: implications for design of future

outcome trials. Br J Anaesth. 2005 ; 94:24-9

4. Rosell PA, Parker MJ. Functional outcome after hip fracture. A 1-year prospective outcome

study of 275 patients. Injury. 2003; 34:529-32

5. Kristensen MT, Foss NB, Kehlet H. [Timed Up and Go and New Mobility Score as predictors of

function six months after hip fracture]. Ugeskr Laeger.2005 ; 167:3297-300

6. Handoll H, Sherrington C. Mobilisation strategies after hip fracture surgery in adults. Cochrane

Database Syst Rev.2007; CD001704

7. Foss NB, Kristensen MT, Kristensen BB, Jensen PS, Kehlet H. Effect of postoperative epidural

analgesia on rehabilitation and pain after hip fracture surgery: a randomized, double-blind,

placebo-controlled trial. Anesthesiology.2005 ; 102:1197-204

8. Foss NB, Kristensen MT, Jensen PS , et al. The effects of liberal versus restrictive transfusion

thresholds on ambulation after hip fracture surgery. Transfusion. 2009; 49:227-34

9. Kristensen MT, Foss NB, Kehlet H. Timed "Up & Go" test as a predictor of falls within 6 months

after hip fracture surgery. Phys Ther. 2007; 87:24-30

10. Kristensen MT, Bandholm T, Bencke J, Ekdahl C, Kehlet H. Knee extension strength, postural

control and function are related to fracture type and thigh edema in patients with hip fracture. Clin

Biomech (Bristol, Avon). 2009; 24:218-24

11. Peterson MD, Rhea MR, Sen A, Gordon PM. Resistance exercise for muscular strength in older

adults: a meta-analysis. Ageing Res Rev. 2010; 9:226-37

12. Mitchell SL, Stott DJ, Martin BJ, Grant SJ. Randomized controlled trial of quadriceps training

after proximal femoral fracture. Clin Rehabil. 2001; 15:282-90

13. Hauer K, Specht N, Schuler M, Bartsch P, Oster P. Intensive physical training in geriatric patients

after severe falls and hip surgery. Age Ageing. 2002; 31:49-57

14. Binder EC, Brown M, Sinacore DR et al. Effects of extended outpatient rehabilitation after hip

fracture: a randomized controlled trial. JAMA. 2004; 292:837-46

15. Kortebein P, Ferrando A, Lombeida J, Wolfe R, Evans WJ. Effect of 10 days of bed rest on

skeletal muscle in healthy older adults. JAMA.2007; 297:1772-4

16. Kortebein P, Symons TB, Ferrando A, et al. Functional impact of 10 days of bed rest in healthy

older adults. J Gerontol A Biol Sci With Sci. 2008; 63:1076-81

17. Suetta C, Magnusson SP, Rosted A et al. Resistance training in the early postoperative phase

reduces hospitalization and leads two muscle hypertrophy in elderly hip surgery patients - a

controlled, randomized study. J Am Geriatr Soc. 2004, 52:2016-22

18. Foss NB, Kristensen MT, Kehlet H. Prediction of postoperative morbidity, mortality and

rehabilitation in hip fracture patients: the cumulated ambulation score. Clin Rehabil. 2006;

20:701-8

19. Kristensen MT, Andersen L, Bech-Jensen R, et al. High inter-tester reliability of the cumulated

ambulation score for the evaluation of basic mobility in patients with hip fracture. Clin Rehabil.

2009;23:1116-23

20. Parker MJ, Palmer CR. A new mobility score for predicting mortality after hip fracture. J Bone

Joint Surg Br. 1993; 75:797-8

21. Burton LA, Sumukadas D. Optimal management of sarcopenia. Clinical Interventions in Aging.

2010; 5:217-28

22. Madsen OR, Lauridsen UB. Knee extensor and flexor strength in elderly women after recent hip

fracture: assessment by the Cybex 6000 dynamometer of intra-rates inter-test reliability. Scand J

Rehabil Med. 1995; 27:219-26

23. Kristensen MT, Foss NB, Ekdahl C, Kehlet H. The prefracture functional level evaluated by the

New Mobility Score predicts in-hospital outcome after hip fracture surgery. Acta Orthop. 2010;

81:296-302

24. Kristensen MT, Bandholm T, Foss NB, Ekdahl C, Kehlet H. High inter-tester reliability of the

New Mobility Score in patients with hip fracture. J Rehabil Med. 2008; 40:589-91

25. Podsiadlo D, Richardson S. The timed "Up & Go": a test of basic functional mobility for frail

elderly persons. J Am Geriatr Soc. 1991; 39:142-8

26. Kristensen MT, Bandholm T, Holm B, Ekdahl C, Kehlet H. Timed Up & Go test score in patients

with hip fracture is related to the type of walking aid. Arch Phys With Rehabil. 2009; 90:1760-5

27. Kristensen MT, Ekdahl C, Kehlet H, Bandholm T. How many trials are needed to achieve

performance stability of the Timed Up & Go test in patients with hip fracture? Arch Phys With

Rehabil. 2010; 91:885-9

**Lay Summary**All patients undergoing surgery for a hip fracture has a need for rehabilitation in an attempt to regain former skills. Despite the optimization of rehabilitation during hospitalization and subsequent outpatient rehabilitation program, it has been shown that some patients are at high risk of falling again, not achieve the same level of physical function and to be long-term dependent on assistant care. Furthermore, it is shown that patients with a hip fracture, within 2 weeks after the fracture, have lost more than half of the muscular strength in the operated limb as compared to the healthy limb. Recent studies with patients who had a hip replacement, where strength training was implemented immediately after the operation as part of the rehabilitation, has shown good effect of this, but no similar studies of patients with an acute hip fracture has been found. This study will *(1)* study the feasibility of strength training (40 patients), and *(2)* by lot, randomize the 100 patients acutely admitted to Hvidovre Hospital with a hip fracture, into two groups. Patients in one group will receive rehabilitation after normal standards during hospitalization, while patients in the other group also will have to perform strengthening exercises for thigh muscles every other day during in-hospital stay. Strength exercises are performed seated, and depending on the individual strength level using with weight cuffs attached to the ankle. No side effects of strength training have been reported, apart from a possible light muscle soreness after the first sessions, corresponding to that experienced by healthy people when starting for example badminton after a summer break.

In addition, strength exercises are already used as part of the treatment for this patient group, but not systematically as planned in this study. Patients participating in the study will, as well as the treating physicians and physical therapists, know which group they are in and they will therefore be able to respond to any issues. Patients are evaluated on a daily basis during hospitalization regarding their ability to participate in the rehabilitation and will otherwise receive treatment according the normal standards of the department. The study spans the period of hospitalization after an acute hip fracture including a 16. week post operative control. It is estimated that patients in the intervention group will experience a reduced loss of muscle strength in the operated limb, with the result that fewer people will experience new fall episodes and that more patients will recover a level of physical function as before the hip fracture. The study may prove of importance for the participants, but also further on for the many other patients with a hip fracture worldwide.
Patients are included after obtaining informed written consent from the patient himself after a minimum of 24 hours of consideration. The study is part of the "Hip fracture project" at Hvidovre Hospital and has an expected duration of 30 months. It should be emphasized that the project is not funded by the pharmaceutical industry and none of the participants have any financial or other interests in the study.
**Patient Information - Purpose and Course of study
Project title**: Physical therapy including progressive resistance training immediately after surgery for hip fracture: Can it be done and what is the effect?

We contact you to ask you to participate in our scientific research. The background of the study is that surgery after hip fracture often leads to many patients permanently getting a lower level of physical function and need of assistive care. Recent studies in patients with a hip fracture, whom as part of the training performed strength exercises, have shown that these patients achieved a higher level of physical function and were less dependent on assistant aid, but these studies started at a later stage of the rehabilitation program after discharge from the orthopedic department where they had surgery. At this late stage, it is shown that patients have lost more than half of their muscle strength in the operated limb.

Thus, there is little evidence to suggest that patients with hip fractures, could have beneficial effect of strengthening exercises in addition to the normal rehabilitation and it is estimated, that a greater number of patients will be able to achieve a level of physical function as before the hip fracture. However, there are no studies documenting the effect of strengthening exercises if you start training immediately after surgery, so the study may have important implications for both the participants in this study, as well as many other future patients with a hip fracture worldwide.

No side effects of strength training have been reported, apart from possible light muscle soreness after first sessions. In addition, strength exercises are already used as part of the treatment for this patient group, but not in such systematized form as planned in this study. If you participate in the study you will along with 100 other patients undergoing surgery for a hip fracture be placed in one of two groups chosen by lot. One group is trained with physical therapists according to normal standards. The second group will be trained by normal standards supplemented by strengthening exercises every day during hospitalization. The only difference in treatment will therefore be that you will not receive strengthening exercises if you are placed in the first group.

 If you participate in the study you will know yourself, as well as treating physicians and physiotherapists, which group you are in, and therefore be able to respond to any issues.

As part of the study you will be assessed on a daily basis during hospitalization regarding your ability to participate in rehabilitation. The rehabilitation continues as for all other patients with a standard outpatient rehabilitation course in the municipality after discharge. You will be invited to a control examination 16 weeks after surgery in the same department where you were hospitalized.

You will in all aspects be treated according to the normal standards in the unit.

If needed, you can always contact the project manager for more information about the project and you are encouraged to read the attached note "Your Rights as a test subject in a biomedical research."

The project is organized by Senior Researcher Morten Tange Kristensen (project manager) in collaboration with Professor Henrik Kehlet and Senior Researcher Thomas Bandholm, with the head of the orthopedic hip fracture unit Henrik Palm as clinically responsible.

None of the potential financial sponsors to the project will have any impact or financial interest in the organization, implementation and interpretation of the data collected in the project.

The study is approved by the Ethical Committee of the Capital Region, Journal number; HA-2007-0127 + Additional Protocol of 31.08.2010 and approved by the Danish Data Protection Agency, Journal number; 2007-41-1573. The study is part of the "Hip-fracture project" at Hvidovre Hospital.

Best Regards

Project Manager/Contact Clinical responsible

Morten Tange Kristensen, Henrik Palm, MD

Senior Researcher, PhD Orthopaedic Surgery Dept. 333

Physiotherapy Dept. 236 Hvidovre Hospital, RegionH

Hvidovre Hospital, RegionH

Tel.: +45 3862 61 91 & +45 26 15 24 33

E-mail: [morten.tange.kristensen@hvh.regionh.dk](mailto:morten.tange.kristensen@hvh.regionh.dk) mail to: [hpalm@dadlnet.dk](mailto:hpalm@dadlnet.dk)

# Patient Information - General ethical and legal requirements

(Attached as a supplement to the "Your Rights as a test subject in a biomedical research”)

This page describes general requirements for studies of this kind that also comply with the current study.

It is entirely optional whether you want to participate in the study or not. Participation is completed only after you have received both written and oral information, and after giving your written consent. Even if you decide to participate, you can later choose to withdraw from the study without having to explain why. If you choose not to participate in the study it will not affect your current or future treatment.

You can request time for consideration and you are entitled to a counselor to go through the oral and written information with you. As the study is scheduled to start the day after surgery, this time of consideration can only be of a limited length.

If you, during the investigation should experience serious unforeseen side effects, or if you because of any other health related issues or practical reasons couldn’t complete the study program, you will be excluded from the study. The study as a whole will be terminated if serious side effects, risks or complications are found, that the study is considered ethically irresponsible to continue.

All information about you and your health is covered by professional confidentiality. The material containing personal information is kept anonymous in a way that it is accessible only to staff in the research group. Data including the identification of subjects will be destroyed at completion of the study.

You have a right to access to the protocol for public Act, which means that you can gain insight to the parts of the study not containing personal information about any other participant.

As a participant in the study are you as any other patient covered by Hvidovre Hospital patient insurance, and you can turn to the patient insurance if you wish to seek compensation. Furthermore, if you wish, you can complain about the treatment through the Patient Complaints Board.

**The subject's rights in a biomedical research project** (attached to the written patient information)

As a participant in a biomedical research study you should know that:

· Your participation in the research project is entirely voluntary and can only be done after you have received both written and oral information about the research project and signed the consent form

· You at any time, orally, in writing or in another clear indication, may withdraw your consent to participate and withdraw from the research project. If you withdraw your consent it will not affect your right to present or future treatment or other rights that you may have

· You have the right to bring a family member, friend or acquaintance to the information interview

· You are entitled to time for consideration before you sign the consent form

· Information about your health, other personal or confidential information about you that might appear in relation with the research project, covered by professional confidentiality

· Storing of information about you, including information in your samples of blood and tissue, comply with the law of Act on Processing of Personal Data and Health Act

· It is possible to gain access to experimental protocols according to Public Records Act. This means that you can get access to view all documents pertaining to your participation in the trial, except for the parts that contain trade secrets or confidential information about other participants

· It is possible to complain and get compensation under the Act on the Complaint and Compensation in public healthcare

Informed consent for participation in a biomedical research project.

Research Project Title:

Physiotherapy including progressive resistance training started immediately after surgery for hip fracture: Can it be done and what is the effect?

Statement by the subject:

I have received written and oral information and I know enough about the objectives, methods, advantages and disadvantages to say yes to participate.

I know that it is voluntary to participate and that I may withdraw my consent without losing my present or future rights to treatment.

I consent to participate in the research project and have received a copy of this consent form and a copy of the written information on the project for personal own use.

The subject's name ______________________________

________Date___________________________________________________________ Signature

Statement by the investigator:

I declare that oral information about the project has been provided, written information handed out, and that the consent on participation in the trial has been obtained from the subject.

The Investigator's name: _____________________________________

________Date___________________________________________________________ Signature

 
