# Supplementary material for: Feasibility of Progressive Strength Training Implemented in the Acute Ward after Hip Fracture Surgery
Source: PLoS One. 2014 Apr 3;9(4):e93332. doi: 10.1371/journal.pone.0093332 (PMC3974729; doi:10.1371/journal.pone.0093332)
Supplement: Protocol S2 — Complete study trial protocol as approved by ethics committee, original language. (DOCX) [file pone.0093332.s002.docx]

Fysioterapi inklusiv progressiv styrketræning opstartet tidligt efter operation for hoftebrud:

Kan det lade sig gøre og med hvilken effekt

#### Investigator og projektleder

Morten Tange Kristensen, Seniorforsker, ph.d., Fysioterapien 236 og Ortopædkirurgisk afdeling 333, RegionH, Hvidovre Hospital, tlf. 38 62 61 91,

E-mail: morten.tange.kristensen@regionh.dk

#### Klinisk ansvarlig

Henrik Palm, Overlæge, Ortopædkirurgisk afdeling 333, RegionH, Hvidovre Hospital,

E-mail: hpalm@dadlnet.dk

#### Øvrige investigatorer

Henrik Kehlet, Professor, ph.d., Sektion for Kirurgisk Patofysiologi 4074, RegionH, Rigshospitalet.

Thomas Bandholm, Seniorforsker, ph.d., Klinisk Forskningsenhed 136, Ortopædkirurgisk Afdeling 247 og Fysioterapien 236, RegionH, Hvidovre Hospital.

#### Undersøgelsessted

Hvidovre Hospital, Kettegård Alle 30, 2650 Hvidovre, tlf. 36 32 36 32

[Protokolresumé 2](#_Toc272302087)

[Formål med undersøgelsen 2](#_Toc272302088)

[Primære formål 2](#_Toc272302089)

[Baggrund 2](#_Toc272302090)

[Undersøgelsesdesign 2](#_Toc272302091)

[Undersøgelsestype 2](#_Toc272302092)

[Randomisering 2](#_Toc272302093)

[Blinding 2](#_Toc272302094)

[Tidsplan 2](#_Toc272302095)

[Afbrydelse 2](#_Toc272302096)

[Dataregistrering 2](#_Toc272302097)

[Patientpopulation 2](#_Toc272302098)

[Inklusionskriterier 2](#_Toc272302099)

[Eksklusionskriterier 2](#_Toc272302100)

[Procedurer 2](#_Toc272302101)

[Standard for træning under indlæggelsen 2](#_Toc272302102)

[Fysioterapi inklusiv styrketræning 2](#_Toc272302103)

[Fysioterapi uden styrketræning 2](#_Toc272302104)

[Efter udskrivning 2](#_Toc272302105)

[Registreringer 2](#_Toc272302106)

[Primære effektmål 2](#_Toc272302107)

[Sekundære effektmål 2](#_Toc272302108)

[Øvrige parametre 2](#_Toc272302109)

[Sikkerhed 2](#_Toc272302110)

[Sikkerhedsvurdering 2](#_Toc272302111)

[Uddannelse 2](#_Toc272302112)

[Etik 2](#_Toc272302113)

[Indhentelse af samtykke 2](#_Toc272302114)

[Databehandling og dataanalyse 2](#_Toc272302115)

[Økonomiske forhold 2](#_Toc272302116)

[Publikation 2](#_Toc272302117)

[Lægmandsresumé 2](#_Toc272302118)

[Patientinformation - Formål og Forløb af Undersøgelsen 2](#_Toc272302119)

[Patientinformation - Generelle etiske aspekter og lovmæssige krav 2](#_Toc272302120)

[Forsøgspersonens rettigheder i et biomedicinsk forskningsprojekt (vedhæftes den skriftlige patientinformation) 2](#_Toc272302121)

# Protokolresumé

Ældre patienter med hoftebrud er en særligt udsat gruppe med høj morbiditet og mortalitet^1-3^ og en del patienter genvinder ikke tidligere funktionsniveau^4,5^. Det seneste Cochrane review, der vurderer effekt af fysioterapi, konkluderer at der er utilstrækkelig evidens fra randomiserede studier til at fastslå effektiviteten af forskellige træningsformer, der anvendes i rehabiliteringen efter hoftebrud kirurgi^6^. Patienter med hoftebrud indlagt på Hvidovre Hospital er siden september 2002 behandlet efter konceptet accelereret operationsforløb^7,8^ i en specialenhed på Ortopædkirurgisk Afdeling. Behandlingen er optimeret og intensiveret indenfor kirurgi, ernæring, smertebehandling, fysioterapi etc. under indlæggelsen. Til trods for den optimerede indsats er patienter, der genudskrives direkte til egen bolig efter at have fulgt et standardforløb, i gennemsnit indlagt i 16 (SD 14) dage og en del udskrives til yderligere rehabilitering på døgnbasis i primærsektoren. Et studie fra enheden har vist at 32 % af patienterne falder igen indenfor 6 måneder efter udskrivning^9^, mens andre data indsamlet i enheden har vist, at muskelstyrken i forreste lårmuskel på det opererede ben blandt patienter, der udskrives direkte til egen bolig udgør mindre end 50 % af styrken i det raske ben^10^, til trods for at de har fulgt et optimeret operationsforløb. Til trods for at der ikke er fundet sikker evidens for at styrketræning på langt sigt er effektivt for patienter med hoftebrud^6^ vurderes det som en væsentlig komponent i rehabiliteringsforløbet. Det er nemlig velkendt, at styrketræning øger muskelstyrken hos raske ældre personer^11^ og patienter med hoftebrud, hvor styrketræning dog tidligst er opstartet 16 dage efter operation^12^. Hertil kommer to andre studier, hvor styrketræning er opstartet senere i forløbet^13,14^. Alle tre studier viste effekt af styrketræning ved afslutning af intervention sammenlignet med kontrolgruppen, men enten var der for få deltagere eller for kort follow-up til at sige noget endegyldigt om effekten af styrketræning som intervention, ligesom interventionen er iværksat så sent, at tab af muskelfunktion allerede er iværksat. Idet man som ældre oplever et gennemsnitligt fald i muskelstyrke på 15% ved at blive lagt i en seng 10 dage^15,16^ og i forvejen har en lav muskelstyrke på grund af høj alder, synes det oplag at starte styrketræning op tidligt hos patienter med hoftebrud for at undgå et stort styrke- funktionstab. Eksempelvis er der blandt elektive patienter opereret med total hoftealloplastik fundet effekt af styrketræning opstartet umiddelbart efter operation^17^. Det vides således ikke om ældre patienter med hoftebrud kan gennemføre fysioterapi inklusiv styrketræning opstartet tidligt efter operation og om effekten er større end fysioterapi uden styrketræning.

Vi ønsker derfor at udføre (1) en undersøgelse af muligheden for at gennemføre styrketræning opstartet dagen efter operation, samt (2) en randomiseret undersøgelse med opstart dagen efter operation, hvor 100 ældre patienter med hoftebrud, der indlægges i Hoftebrudenheden på Hvidovre Hospital randomiseres til fysioterapi med eller uden progressiv styrketræning.

Undersøgelsen der er en del af ”Hoftefraktur projektet”, Ortopædkirurgisk Afdeling, Hvidovre Hospital, påregnes at strække sig over en periode på 30 måneder. Det skal pointeres at undersøgelsen ikke er støttet af eller har tilknytning til medicinalindustrien, ligesom ingen af deltagerne har økonomisk eller anden interesse i undersøgelsen.

## Formål med undersøgelsen

### Primære formål

At undersøge (1) muligheden for at gennemføre styrketræning i den tidlige postoperative fase efter hoftebrud, (2) effekten af fysioterapi med og uden progressiv styrketræning i den tidlige postoperative fase efter hoftebrud.

## Baggrund

Patienter med hoftebrud er en særligt udsat gruppe med høj morbiditet og mortalitet^1-3^ og mange patienter genvinder ikke tidligere funktionsniveau^4,5^.

I det accelererede multimodale interventionsprogram for patienter med hoftebrud^7^ som alle patienter på Hvidovre Hospital følger, fokuseres der på tidlig operation, forebyggelse af perioperativ hypoksæmi, optimeret perioperativ analgesi, adækvat ernæring og væsketerapi samt tidlig mobilisering og fysioterapi. I dette regime bliver patienterne mobiliseret og modtager fysioterapi alle ugens 7 dage (første 3 dage efter operation, herefter kun på hverdage). På afdelingen er der udviklet et nyt scoringssystem, the Cumulated Ambulation Score (CAS)^18,19^, der beskriver niveau for basismobilitet, defineret som evnen til at komme ud-ind af seng, op-ned af stol og gang med et egnet gangredskab. CAS-scoren for de første 3 dage efter operation har for patienter indlagt fra egen bolig, vist sammenhæng med; udskrivning inden for 14 dage, udskrivning til oprindelig bolig, 30-dages mortalitet og medicinske komplikationer. Et seks måneders opfølgningsstudie^5^ udgået fra samme enhed, har vist at der selv blandt de bedste af patienterne fortsat var 70 %, der anvendte et gangredskab mod kun 40 % før bruddet og at 32 % havde oplevet et eller flere fald efter udskrivning^9^. Samme undersøgelse viste desuden, at det overvejende var patienter med en New Mobility Score (0-9)^20^ beskrivende funktionsniveau før hoftebruddet på ≥ 6 (højt niveau), det var muligt at følge.

Patienterne er karakteriseret ved en høj gennemsnitsalder, der for mange er forbundet med sarcopenia (aldersrelateret tab af muskelmasse, styrke og funktion)^21^ og en forværringen af dette er en kendt følge af et hoftebrud. Data indsamlet i enheden har vist, at muskelstyrke i det opererede ben blandt patienter der udskrives direkte til egen bolig til trods for dette intensive rehabiliteringsforløb udgør mindre end 50 % af styrken i det raske ben^10^, svarende til niveau set i et tidligere studie^22^. Dertil at styrketabet var markant højere blandt patienter med trokantære hoftebrud, sammenlignet med patienter med cervikale hoftebrud, og at graden af styrketab var stærkt associeret med graden af hævelse (ødem) i det opererede ben^10^. Betydningen af frakturtype er yderligere dokumenteret i et studie der viser at sandsynligheden for at opnå selvstændighed i basismobilitet vurderet ved CAS, og blive udskrevet til oprindelig bolig er mindre for patienter med trokantære brud i forhold til dem der har pådraget sig et cervikalt brud^23^. Styrketræning øger muskelstyrken hos raske ældre personer^11^ og tre studier har vist korttidseffekt af styrketræning hos patienter med hoftebrud opstartet i et senere stadie af rehabiliteringsforløbet,^12-14^ samt hos elektive patienter opereret med total hoftealloplastik opstartet umiddelbart efter operation^17^. Det vurderes således relevant at afprøve fysioterapi inklusiv styrketræning, med opstart i den helt tidlige postoperative fase hos patienter med hoftebrud.

## Undersøgelsesdesign

## Undersøgelsestype

Første del: Undersøgelsen af muligheden for at gennemføre styrketræning under indlæggelsen planlægges gennemført med 40 patienter der opfylder inklusionskriterier for det randomiserede studie. Dette projekt færdiggøres forud for iværksættelse af det randomiserede studie.

Randomiseret del af undersøgelsen udføres som en enkeltblindet undersøgelse:

Randomisering

Styrke

Run in

behandlingsperiode Follow up

Kontrol

Test

Test

Screening

Tid ________________________________________________________________________________________

Opr. 1.postopr.dag 10.postopr.dag/udskrivning 16 uger

### Randomisering

Patienterne randomiseres til en af de to behandlingsgrupper. Randomiseringslisten udarbejdes via computer. Ud fra randomiseringslisten laves kuverter med patientnummer på, indeholdende allokering af patient til standard fysioterapi + styrketræning vs. standard fysioterapi uden styrketræning som den foregår i dag med øvelser og funktionstræning. Kuverterne åbnes først for den enkelte patient efter inklusion. Randomiseringslisten opbevares i lukket kuvert, der opbevares af den klinisk ansvarlige. Patienterne stratificeres efter frakturtype, således at der i hver gruppe inkluderes lige mange med cervikale versus inter/subtrokantære brud.

### Blinding

Randomiseret studie: Patienter, læger, plejepersonale og behandlende fysioterapeuter kan af gode grunde ikke være blindede, men test ved inklusion, udskrivning, og 16 uger vil foretages af personer uden kendskab til behandlingsgruppe.

### Tidsplan

Første del: De 40 patienter planlægges inkluderet i perioden medio 2012 - ultimo 2012. Styrketræning viste sig mulig at gennemføre og uden smerter eller andre begrænsende faktorer i forbindelse med dette.

Randomiseret del: Det forventes at påbegynde rekruttering af patienterne medio 2013. Undersøgelsen forventes at strække sig over en periode på cirka 18 måneder, forudsat en forventet inklusion på 6 patienter om måneden.

### Afbrydelse

Den enkelte patient kan vælge at trække sig fra undersøgelsen på et hvilket som helst tidspunkt. Patienterne kan ligeledes udgå af undersøgelsen på et hvilket som helst tidspunkt efter investigators skøn.

### Dataregistrering

Alle for undersøgelsen relevante data registreres på et til formålet udfærdiget skema. Der udfyldes et skema for alle inkluderede patienter.

## Patientpopulation

Randomiseret del: Beregninger har vist, at der for at vise en 30% reduktion i forskellen i styrke mellem opereret og rask ben, med et signifikans niveau på 0,05 og 80 % styrke, skal inkluderes 42 patienter i hver gruppe. Grundet forventet frafald inkluderes der derfor 100 ældre patienter, der indlægges akut fra egen bolig med hoftebrud.

### Inklusionskriterier

- Patienter med en alder på ≥60 år, der indlægges akut under diagnosen hoftebrud (cervikale, pertrokantær eller –subtrokantær fraktur).
- Skal kunne tale og forstå dansk.
- Skal være i stand til at give informeret samtykke selv.
- Hjemmeboende med selvstændig gangfunktion svarende til en New Mobility Score på ≥ 2 indendørs.

### Eksklusionskriterier

- Multiple operationskrævende frakturer
- Postoperativ kirurgisk mobiliseringsrestriktion
- Patient vil ikke deltage i relevant genoptræning
- Terminal lidelse
- Patienter der ønsker en bisidder tilstede ved informationssamtalen, men hvor en sådan ikke har mulighed for at være tilstede

## Procedurer

Første del: Styrketræningsforløb der er en mulig del af det fysioterapeutiske tilbud i dag, forsøges gennemført med opstart dag 1. eller 2. efter operation. Målet er styrketræning hver dag under indlæggelsen.

Randomiseret del:

1. Information og afgivelse af informeret samtykke, inden eller senest på 2. postoperative dag efter indlæggelse.
2. Randomisering til to grupper af 50 patienter ved projektleder eller en af de øvrige investigatorer.

## Standard for træning under indlæggelsen

### Fysioterapi inklusiv styrketræning

Træner dagligt på hverdage (første 3 dage efter operation også weekend) under indlæggelsen med fysioterapeut med fokus på selvstændighed i basale aktiviteter (samme program som gruppe, fysioterapi uden styrketræning) samt:

Opereret ben trænes med knæekstensionsøvelser 0-90 grader. Udgangsstillingen er siddende i stol, på seng eller briks med 90 graders fleksion i knæ, koncentrisk og den excentriske bevægelse udføres langsomt og kontrolleret. Træningen udføres med vægtmanchetter.

Der trænes så vidt muligt efter følgende program fra 1. postoperative dag under indlæggelsen:

3 sæt med 10 gentagelser, 10 RM (repetitions maksimum) belastning.

Patienter der på en given dag ikke er i stand til at gennemføre hele programmet, gennemfører så mange sæt og gentagelser som muligt

### Fysioterapi uden styrketræning

Træner "fodvip" i seng og følgende aktiviteter dagligt: ud/ind af seng, rejse/sætte sig i stol, gang med egnet gangredskab og trappegang under hele indlæggelsen.

## Efter udskrivning

Alle patienter følger standard genoptræningstilbud i primærsektor (normalt op til 8 gange på hold)

## Registreringer

### Primære effektmål

#### Fysioterapi og mobilisering

**Under indlæggelse og ved udskrivning**.

Til vurdering af tidligere funktionsniveau anvendes New Mobility Score (NMS)^20,24^. Patienterne vurderes dagligt på deres evne til at udføre basale funktioner, defineret som: ud-ind af seng, op-ned af stol og gang med aktuelle gangredskab. Til beskrivelse af niveau for dette anvendes The Cumulated Ambulation Score^18^. Endvidere registreres tiden for den såkaldte Timed Up and Go test (TUG)^25-27^, og 10 meter gang (fast speed), der vurderer funktionel mobilitet ved udskrivning. Testene er en del af den normale fysioterapeutiske træning på afdelingen. Det vurderes, i tilfælde af manglende evne hos en patient til at udføre styrketræning eller en given funktion selvstændigt, hvilke begrænsende faktorer der gør sig gældende. Lår og – læg omfang for opereret og rask ben måles ved inklusion og ved efterfølgende test, som i tidligere studie^10^.

Isometrisk styrke måles med håndholdt dynamometer så tidligt efter inklusion som muligt.

Alle test/undersøgelser udføres herudover på 10. postoperative dag hvis muligt og/eller dagen før udskrivning.

**16. uger postoperativt.**

TUG, 10 meter gang, NMS, isometrisk styrke for begge ben.

### Sekundære effektmål

#### Indlæggelsestid, og fald

Indlæggelsestiden registreres som værende tiden fra ankomst til hospitalet til det tidspunkt hvor patienten enten er udskrevet eller er død. Dag hvor patienter opfylder udskrivningskriterier, samt dag for indstilling til genoptræningsophold eller plejehjem for patienter der ikke kan udskrives til oprindelige bolig, registreres også. Fald registreres 16 uger efter operation.

For begge grupper registreres træningsforløb under indlæggelse og i ambulant regi, herunder antal besøg og type af træning.

### Øvrige parametre

Vægt og højde

## Sikkerhed

### Sikkerhedsvurdering

Den sikkerhedsmæssige problemstilling er relateret til styrketræning. Studier med styrketræning har ikke beskrevet sikkerhedsmæssige problemer, og det planlagte program sikrer en tilvænningsfase. Ud fra den tilgængelige evidens udsættes patienterne således ikke for nogen sikkerhedsmæssig risiko, udover at der kan forekomme let muskelømhed efter de første træningsgange. Studiet er ikke blindet for behandlerne, hvorfor identifikation af eventuelle sikkerhedsproblemer vil være let.

### Uddannelse

Observationer og målinger foretages af fysioterapeuter, læger og sygeplejersker med tilknytning til projektet.

#### Utilsigtede hændelser

I denne sammenhæng defineres utilsigtede hændelser som ethvert utilsigtet, ugunstigt fund, symptom eller sygdom, der kan tilskrives styrketræning, hvad enten den vurderes at have en sammenhæng med denne eller ej. Utilsigtede hændelser registreres ved spontan registrering såvel som ved åben udspørgning.

#### Alvorlige utilsigtede hændelser

I denne sammenhæng defineres alvorlige utilsigtede hændelser som hændelser eller bivirkninger, der forårsager:

- Død
- Livstruende situationer
- Indlæggelse på hospital eller forlængelse af eksisterende indlæggelse
- Permanent eller svært handicap/uarbejdsdygtighed

Alvorlige utilsigtede hændelser skal af investigator vurderes for mulige sammenhænge med behandlingen i interventionsgruppen, for at overveje om der er en rimelig mulighed for at den utilsigtede hændelse har været forårsaget af denne. Følgende faktorer medtages i vurderingen:

Sammenhæng i tid.

Overensstemmelse med kendte konsekvenser af behandlingen.

Alternative årsager.

Hvis en alvorlig utilsigtet hændelse vurderes at have en kausal sammenhæng med behandlingen skal investigator overveje om undersøgelsen skal afsluttes før tid.

## Etik

Der er ikke fundet studier, der beskæftiger sig med intensiv styrketræning opstartet i den helt tidlige postoperative fase og dets betydning for rehabilitering af patienter med hoftebrud, hvorfor studiet således absolut er relevant, ligesom det, som argumenteret tidligere, ikke bør udgøre nogen sikkerhedsmæssig risiko for patienten. Undersøgelsen vil blive udført i overensstemmelse med principperne i Helsinki deklarationen.

Patienterne er dækket af Hvidovre Hospitals patientforsikring.

Protokollen inklusive patientinformation og samtykkeerklæring for det randomiserede studie er godkendt af den Videnskabsetiske Komité for Region Hovedstaden (H-A-2007-0127) og registreret hos ClinicalTrials.gov.

Investigator er ansvarlig for at informere den Videnskabsetiske Komité om enhver alvorlig utilsigtet hændelse og/eller større ændringer i protokollen. Al korrespondance arkiveres af den koordinerende investigator.

## Indhentelse af samtykke

Alle patienter modtager mundtlig og skriftlig information om undersøgelsen. Inklusion foregår efter opnåelse af informeret samtykke fra patienten selv. Det er investigators ansvar at give patienterne fyldestgørende skriftlig og mundtlig information om undersøgelsens forløb, formål, risici og mulige fordele.

Patienterne kontaktes personligt af projektleder efter indlæggelse på Ortopædkirurgisk afdeling 310, Hvidovre Hospital. Ved rekrutteringen af patienten oplyses at det drejer sig om en videnskabelig undersøgelse, at patienten har ret til at have en bisidder til stede under informationen samt at patienten kan udbede sig en vis betænkningstid efter afgivelse af information***.*** For at sikre at patienten får mindst 24 timers betænkningstid inden eventuel underskrift af samtykkeerklæring, planlægges såvel mundtlig som skriftlig informationen afgivet forud for operation. Dette for at patienter i interventionsgruppen så vidt muligt kan opstarte styrketræning dagen efter operationen. Den mundtlige information, der gives i forbindelse med udlevering af den skriftlige følger indholdet af denne, idet denne gennemgås og de enkelte forhold uddybes. Den mundtlige information tilpasses patientens alder, sociale forhold og uddannelsesniveau. Det sikres at patienten har forstået indholdet i både den mundtlige og skriftlige information. Den mundtlige information gives af den projektansvarlige eller af en anden bemyndiget person i forskningsgruppen. Den projektansvarlige sikrer sig, at en sådan bemyndiget person er grundigt informeret om projektet og fagligt kvalificeret til at afgive informationen. Samtykke til deltagelse i undersøgelsen søges indhentet dagen efter afgivelse af information efter at patienten har haft minimum 24 timers betænkningstid.

## Databehandling og dataanalyse

Patienterne identificeres ved hjælp af et tildelt nummer. Ved undersøgelsens afslutning bliver alle personidentificerbare data destrueret. Patienterne informeres mundtligt og skriftligt om at data bliver opbevaret og analyseret i en computer, at patientens anonymitet bliver bevaret, og at datalovgivningen bliver overholdt. Under forudsætning af normalfordelte data vil den primære analyse være repeated measures variansanalyse (ANOVA) til bestemmelse af systematisk forskel mellem intervention- og kontrolgruppe. Dertil foretages en intention-to-treat analyse. Studiet er godkendt af datatilsynet J.nr. 2007-41-1573.

## Økonomiske forhold

Undersøgelsen er en del af Hvidovre Hospitals ”Hoftefrakturprojekt”. Det skal understreges at undersøgelsen ikke støttes af medicinalindustrien eller af nogle andre institutioner med økonomisk interesse i den undersøgte problematik, ligesom ingen deltagere i projektgruppen har økonomiske eller andre interesser i den undersøgte problematik.

Der vil blive søgt om økonomisk støtte til gennemførsel af undersøgelsen, men ingen af eventuelle støttegivere til projektet, vil have nogen indflydelse eller økonomisk interesse i tilrettelæggelse, gennemførelse og tolkning af de data, der indsamles i projektet.

## Publikation

Undersøgelsens resultater, såvel positive som negative publiceres i et internationalt engelsksproget tidsskrift. Projektlederen udarbejder manuskript, er korresponderende forfatter og står som 1. forfatter, alle investigatorer kommer med som forfattere.

Ligeledes vil resultaterne blive præsenteret på relevante nationale og internationale kongresser. Derudover vil der, forudsat positive erfaringer med styrketræning til patienter med hoftebrud blive arbejdet for at procedure for dette indarbejdes i nationale retningslinjer.

Reference List

1. Rasmussen S, Kristensen BB, Foldager S, Myhrmann L, Kehlet H. [Accelerated recovery program after hip fracture surgery]. Ugeskr Laeger.2002;165:29-33

2. Sharrock NE. Fractured femur in the elderly: intensive perioperative care is warranted. Br J Anaesth.2000;84:139-40

3. Foss NB, Kehlet H. Mortality analysis in hip fracture patients: implications for design of future outcome trials. Br J Anaesth.2005;94:24-9

4. Rosell PA, Parker MJ. Functional outcome after hip fracture. A 1-year prospective outcome study of 275 patients. Injury.2003;34:529-32

5. Kristensen MT, Foss NB, Kehlet H. [Timed Up and Go and New Mobility Score as predictors of function six months after hip fracture]. Ugeskr Laeger.2005;167:3297-300

6. Handoll H, Sherrington C. Mobilisation strategies after hip fracture surgery in adults. Cochrane Database Syst Rev.2007;CD001704

7. Foss NB, Kristensen MT, Kristensen BB, Jensen PS, Kehlet H. Effect of postoperative epidural analgesia on rehabilitation and pain after hip fracture surgery: a randomized, double-blind, placebo-controlled trial. Anesthesiology.2005;102:1197-204

8. Foss NB, Kristensen MT, Jensen PS et al. The effects of liberal versus restrictive transfusion thresholds on ambulation after hip fracture surgery. Transfusion.2009;49:227-34

9. Kristensen MT, Foss NB, Kehlet H. Timed "up & go" test as a predictor of falls within 6 months after hip fracture surgery. Phys Ther.2007;87:24-30

10. Kristensen MT, Bandholm T, Bencke J, Ekdahl C, Kehlet H. Knee-extension strength, postural control and function are related to fracture type and thigh edema in patients with hip fracture. Clin Biomech (Bristol, Avon). 2009;24:218-24

11. Peterson MD, Rhea MR, Sen A, Gordon PM. Resistance exercise for muscular strength in older adults: a meta-analysis. Ageing Res Rev.2010;9:226-37

12. Mitchell SL, Stott DJ, Martin BJ, Grant SJ. Randomized controlled trial of quadriceps training after proximal femoral fracture. Clin Rehabil.2001;15:282-90

13. Hauer K, Specht N, Schuler M, Bartsch P, Oster P. Intensive physical training in geriatric patients after severe falls and hip surgery. Age Ageing.2002;31:49-57

14. Binder EF, Brown M, Sinacore DR et al. Effects of extended outpatient rehabilitation after hip fracture: a randomized controlled trial. JAMA.2004;292:837-46

15. Kortebein P, Ferrando A, Lombeida J, Wolfe R, Evans WJ. Effect of 10 days of bed rest on skeletal muscle in healthy older adults. JAMA.2007;297:1772-4

16. Kortebein P, Symons TB, Ferrando A et al. Functional impact of 10 days of bed rest in healthy older adults. J Gerontol A Biol Sci Med Sci.2008;63:1076-81

17. Suetta C, Magnusson SP, Rosted A et al. Resistance training in the early postoperative phase reduces hospitalization and leads to muscle hypertrophy in elderly hip surgery patients--a controlled, randomized study. J Am Geriatr Soc.2004;52:2016-22

18. Foss NB, Kristensen MT, Kehlet H. Prediction of postoperative morbidity, mortality and rehabilitation in hip fracture patients: the cumulated ambulation score. Clin Rehabil.2006;20:701-8

19. Kristensen MT, Andersen L, Bech-Jensen R et al. High intertester reliability of the cumulated ambulation score for the evaluation of basic mobility in patients with hip fracture. Clin Rehabil.2009;23:1116-23

20. Parker MJ, Palmer CR. A new mobility score for predicting mortality after hip fracture. J Bone Joint Surg Br.1993;75:797-8

21. Burton L.A, Sumukadas D. Optimal management of sarcopenia. Clinical Interventions in Aging.2010;5:217-28

22. Madsen OR, Lauridsen UB. Knee extensor and flexor strength in elderly women after recent hip fracture: assessment by the Cybex 6000 dynamometer of intra-rater inter-test reliability. Scand J Rehabil Med.1995;27:219-26

23. Kristensen MT, Foss N.B., Ekdahl C, Kehlet H. The prefracture functional level evaluated by the New Mobility Score predicts in-hospital outcome after hip fracture surgery. Acta Orthop.2009;Accepted:

24. Kristensen M T, Bandholm T, Foss N B, Ekdahl C, Kehlet H. High inter-tester reliability of the New Mobility Score in patients with hip fracture. J Rehabil Med.2008;40:589-91

25. Podsiadlo D, Richardson S. The timed "Up & Go": a test of basic functional mobility for frail elderly persons. J Am Geriatr Soc.1991;39:142-8

26. Kristensen MT, Bandholm T, Holm B, Ekdahl C, Kehlet H. Timed Up & Go test score in patients with hip fracture is related to the type of walking aid. Arch Phys Med Rehabil.2009;90:1760-5

27. Kristensen MT, Ekdahl C, Kehlet H, Bandholm T. How many trials are needed to achieve performance stability of the Timed Up & Go test in patients with hip fracture? Arch Phys Med Rehabil.2010;91:885-9

## Lægmandsresumé

Patienter der opereres for hoftebrud har alle et behov for genoptræning for om muligt at genvinde tidligere færdigheder. Til trods for en optimering af genoptræningen under indlæggelsen og et efterfølgende ambulant genoptræningsforløb, har det vist sig at en del patienter er i stor risiko for at falde igen, ikke opnår samme funktionsniveau og varigt er mere afhængig af hjælp fra det offentlige. Desuden er det vist, at patienter med hoftebrud indenfor 2 uger efter bruddet, har mistet mere end halvdelen af muskelstyrken i det opererede ben i forhold til det raske ben***.*** Nyere undersøgelser med patienter der har fået indsat en ny hofte, hvor styrketræning opstartet umiddelbart efter operation har indgået som en del af genoptræningen, har vist god effekt af dette, men der er ikke fundet lignende undersøgelser for patienter med akut hoftebrud. Denne undersøgelse vil (1) undersøge muligheden for at gennemføre styrketræning (40 patienter), og (2) ved lodtrækning, opdele 100 patienter der indlægges akut på Hvidovre Hospital med hoftebrud, i to grupper. Patienterne i den ene gruppe vil modtage genoptræning efter normale standarder under indlæggelsen, mens patienterne i den anden gruppe udover dette vil skulle udføre styrkeøvelser for lårmuskulatur hver dag. Styrkeøvelserne, der udføres siddende, vil afhængigt af den enkeltes styrkeniveau blive udført med vægtmanchetter der fastgøres om anklerne. Der er ikke konstateret nogen bivirkninger ved styrketræning, udover at der kan forekomme let muskelømhed i starten, svarende til det raske personer oplever ved opstart af for eksempel badminton efter en sommerpause. Desuden anvendes styrkeøvelser allerede som en del af behandlingen til denne patientgruppe, dog ikke i så systematiseret form som planlagt i dette studie. Patienterne der deltager i undersøgelsen vil som de behandlende læger og fysioterapeuter vide hvilken gruppe de er i og de vil derfor være i stand til at reagere på eventuelle problemstillinger. Patienterne vurderes dagligt under indlæggelsen for deres evne til at deltage i genoptræningen og behandles i øvrigt på alle måder efter afdelingens normale standarder. Undersøgelsen strækker sig over den periode patienten er indlagt efter sit hoftebrud, med opfølgende test/kontrol 16 uger efter operation. Det vurderes at patienterne i interventionsgruppen vil opleve et mindre tab af muskelstyrke i det opererede ben, med det resultat at færre vil opleve nye fald og at flere generhverver et funktionsniveau som før hoftebruddet. Undersøgelsen kan således vise sig at have stor betydning for deltagerne, men fremadrettet også for de mange andre patienter med hoftebrud på verdensplan.

Patienterne inkluderes efter opnåelse af informeret skriftligt samtykke fra patienten selv efter minimum 24 timers betænkningstid. Undersøgelsen er en del af ”Hoftefrakturprojektet” på Hvidovre Hospital og påregnes at strække sig over en periode på 30 måneder.

Det skal pointeres at projektet ikke er støttet af medicinalindustrien ligesom ingen af deltagerne har økonomiske eller andre interesser i undersøgelsen.

**Patientinformation** **- Formål og Forløb af Undersøgelsen**

**Projektets titel:** Fysioterapi inklusiv progressiv styrketræning opstartet tidligt efter operation for hoftebrud: Kan det lade sig gøre og med hvilken effekt

Vi henvender os til dig for at spørge om du vil være med i vores videnskabelige forskningsprojekt. Baggrunden for undersøgelsen er, at operation for hoftebrud ofte fører til at mange personer varigt får et lavere funktionsniveau med behov for mere hjælp fra det offentlige. Nyere undersøgelser med patienter med hoftebrud der som en del af træningen udførte styrkeøvelser har vist at disse patienter fik et højere funktionsniveau og var mindre afhængige af hjælp, men disse undersøgelser startede først i et senere stadie af rehabiliteringsforløbet efter udskrivning fra den ortopædkirurgiske afdeling hvor de var blevet opereret. På dette sene tidspunkt er det vist at patienter har mistet mere end halvdelen af muskelstyrken i det opererede ben.

Der er således meget der tyder på at patienter med hoftebrud, kunne have gavnlig effekt af styrkeøvelser som supplement til den normale genoptræning og det vurderes at en større andel vil kunne opnå et funktionsniveau som før hoftebruddet. Der eksisterer dog ingen undersøgelser der dokumenterer effekt af styrkeøvelser hvis man starter træningen lige efter operationen, hvorfor undersøgelsen kan have stor betydning for, såvel deltagerne i denne undersøgelse, men fremadrettet også for de mange andre patienter med hoftebrud på verdensplan.

Der er ikke konstateret bivirkninger ved styrkeøvelser udover at der kan forekomme let muskelømhed efter de første gange. Desuden anvendes styrkeøvelser allerede som en del af behandlingen til patienter indlagt i hoftebrudenheden på Hvidovre Hospital, dog ikke i så systematiseret form som planlagt i dette studie. Hvis du deltager i undersøgelsen vil du sammen med 100 andre patienter, der opereres for hoftebrud, ved lodtrækning blive placeret i en af to grupper. I den ene gruppe trænes med fysioterapeuten efter normale standarder. I den anden gruppe trænes efter normale standarder, suppleret med styrkeøvelser hver anden dag under indlæggelsen. Den eneste forskel i behandlingen vil således være at du ikke vil modtage styrkeøvelser hvis du placeres i den ene gruppe.

Hvis du deltager i undersøgelsen vil du således selv vide hvilken gruppe du er i, de behandlende læger og fysioterapeuter vil også vide dette og vil derfor være i stand til at reagere på eventuelle problemstillinger.

Som en del af undersøgelsen vurderes du dagligt under indlæggelsen for din evne til at deltage i genoptræningen. Genoptræningen fortsætter som for andre patienter, med et standard ambulant forløb i kommunen efter udskrivningen. Du vil blive indkaldt til en kontrol 16 uger efter operation på samme afdeling hvor du var indlagt.

Du vil i øvrigt på alle måder blive behandlet efter afdelingens normale standarder.

Ved behov, kan du altid henvende dig til projektlederen for at få yderligere information om projektet og du opfordres til at læse det vedhæftede notat "Dine rettigheder som forsøgsperson i et biomedicinsk forskningsprojekt".

Projektet er tilrettelagt af Seniorforsker Morten Tange Kristensen (projektansvarlig) i samarbejde med Professor Henrik Kehlet og Seniorforsker Thomas Bandholm, med ortopædkirurgisk Overlæge Henrik Palm som klinisk ansvarlig.

Ingen af potentielle støttegiverne til projektet, vil have nogen indflydelse eller økonomisk interesse i tilrettelæggelse, gennemførelse og tolkning af de data, der indsamles i projektet.

Den randomiserede del af undersøgelsen er godkendt af Den Videnskabsetiske komité for region hovedstaden som Journal-nummer; H-A-2007-0127 + tillægsprotokol af 31.08.2010, og er godkendt af Datatilsynet som Journal-nummer; 2007-41-1573. Undersøgelsen er en del af ”Hoftefrakturprojektet” på Hvidovre Hospital.

Med Venlig Hilsen

Projektleder/Kontaktperson Klinisk ansvarlig

Morten Tange Kristensen, Henrik Palm, Overlæge

Seniorforsker, PhD Ortopædkirurgisk afd. 333

Fysioterapien afd. 236 Hvidovre Hospital, RegionH

Hvidovre Hospital, RegionH

Tlf.: 38 62 61 91 & 26 15 24 33

E-mail: [morten.tange.kristensen@hvh.regionh.dk](mailto:morten.tange.kristensen@hvh.regionh.dk) E-mail to: [hpalm@dadlnet.dk](mailto:hpalm@dadlnet.dk)

## Patientinformation - Generelle etiske aspekter og lovmæssige krav

***(vedhæftes som supplement til "dine rettigheder som forsøgsperson i et biomedicinsk forskningsprojekt”)***

På denne side er der beskrevet generelle krav til undersøgelser af denne slags, der også gør sig gældende i den aktuelle undersøgelse.

Det er fuldstændigt ***frivilligt*** om du vil være med i undersøgelsen eller ej. Deltagelse sker kun efter at du har modtaget både skriftlig og mundtlig information, og du har afgivet dit skriftlige samtykke. Selv om du skulle beslutte at være med, kan du ***altid*** senere vælge at trække dig ud af undersøgelsen, uden at du behøver at forklare hvorfor. Hvis du vælger ikke at være med i undersøgelsen vil det ikke påvirke din nuværende eller fremtidige behandling.

Du kan udbede dig betænkningstid, og du har ret til sammen med en bisidder at få gennemgået den mundtlige og skriftlige information. Da undersøgelsen er planlagt til at starte dagen efter du er blevet opereret, kan denne betænkningstid dog kun være af begrænset længde.

Hvis du under undersøgelsen skulle få alvorlige uforudsete bivirkninger, eller hvis du af anden sygdomsmæssige eller praktiske årsager ikke kan gennemføre undersøgelsesprogrammet, vil du blive udelukket fra undersøgelsen. Undersøgelsen som helhed vil blive afbrudt, hvis der viser sig bivirkninger, risici eller komplikationer, der er så alvorlige, at det vurderes etisk uforsvarligt at fortsætte.

Alle oplysninger om dig og dine helbredsforhold er omfattet af tavshedspligt. Materialet indeholdende personlige oplysninger opbevares anonymiseret således at det udelukkende er tilgængeligt for personalet i forskningsgruppen. Data der muliggør identificering af forsøgspersoner bliver destrueret ved undersøgelsens afslutning.

Du har ret til aktindsigt i forsøgsprotokollen efter offentlighedslovens regler, hvilket vil sige at du kan få indsigt i de dele af undersøgelsen, der ikke indeholder personlige oplysninger om andre.

Som deltager i undersøgelsen er du på linje med andre patienter dækket af Hvidovre Hospitals patientforsikring, og du kan henvende dig til patientforsikringen, hvis du ønsker at søge erstatning. Endvidere kan du hvis du ønsker det, klage over behandling gennem Patientklagenævnet.

## Forsøgspersonens rettigheder i et biomedicinsk forskningsprojekt (vedhæftes den skriftlige patientinformation)

Som deltager i et biomedicinsk forskningsprojekt skal du vide at:

- din deltagelse i forskningsprojektet er helt frivillig og kan kun ske efter, at du har fået både skriftlig og mundtlig information om forskningsprojektet og underskrevet samtykkeerklæringen
- du til enhver tid mundtligt, skriftligt eller ved anden klar tilkendegivelse kan trække dit samtykke til deltagelse tilbage og udtræde af forskningsprojektet. Såfremt du trækker dit samtykke tilbage påvirker dette ikke din ret til nuværende eller fremtidig behandling eller andre rettigheder, som du måtte have
- du har ret til at tage et familiemedlem, en ven eller en bekendt med til informationssamtalen
- du har ret til betænkningstid, før du underskriver samtykkeerklæringen
- oplysninger om dine helbredsforhold, øvrige rent private forhold og andre fortrolige oplysninger om dig, som fremkommer i forbindelse med forskningsprojektet, er omfattet af tavshedspligt
- opbevaring af oplysninger om dig, herunder oplysninger i dine blodprøver og væv, sker efter reglerne i lov om behandling af personoplysninger og sundhedsloven
- der er mulighed for at få aktindsigt i forsøgsprotokoller efter offentlighedslovens bestemmelser. Det vil sige, at du kan få adgang til at se alle papirer vedrørende din deltagelse i forsøget, bortset fra de dele, som indeholder forretningshemmeligheder eller fortrolige oplysninger om andre
- der er mulighed for at klage og få erstatning efter reglerne i lov om klage- og erstatningsadgang inden for sundhedsvæsenet

**Informeret samtykke til deltagelse i et biomedicinsk forsknings-projekt.**

Forskningsprojektets titel:

**Fysioterapi inklusiv progressiv styrketræning opstartet tidligt efter operation for hoftebrud: Kan det lade sig gøre og med hvilken effekt**

Erklæring fra forsøgspersonen:

Jeg har fået skriftlig og mundtlig information og jeg ved nok om formål, metode, fordele og ulemper til at sige ja til at deltage.

Jeg ved, at det er frivilligt at deltage, og at jeg altid kan trække mit samtykke tilbage uden at miste mine nuværende eller fremtidige rettigheder til behandling.

Jeg giver samtykke til, at deltage i forskningsprojektet og har fået en kopi af dette samtykkeark samt en kopi af den skriftlige information om projektet til eget brug.

Forsøgspersonens navn ______________________________

________Dato                                             Underskrift

Erklæring fra den forsøgsansvarlige**:**

Jeg erklærer, at der er givet mundtlig information om projektet, udleveret skriftlig information, og der foreligger et samtykke til, at forsøgspersonen kan deltage.

Den forsøgsansvarliges navn: _____________________________________

________Dato                                             Underskrift
